# Tn*6188* - A Novel Transposon in *Listeria monocytogenes* Responsible for Tolerance to Benzalkonium Chloride

**DOI:** 10.1371/journal.pone.0076835

**Published:** 2013-10-02

**Authors:** Anneliese Müller, Kathrin Rychli, Meryem Muhterem-Uyar, Andreas Zaiser, Beatrix Stessl, Caitriona M. Guinane, Paul D. Cotter, Martin Wagner, Stephan Schmitz-Esser

**Affiliations:** 1 Institute for Milk Hygiene, University of Veterinary Medicine Vienna, Vienna, Austria; 2 Christian Doppler Laboratory for Molecularbiological Food Analytics, University of Veterinary Medicine, Vienna, Austria; 3 Teagasc Food Research Centre, Moorepark, Fermoy, Cork, Ireland; 4 Alimentary Pharmabiotic Centre, University College Cork, Cork, Ireland; Cornell University, United States of America

## Abstract

Controlling the food-borne pathogen *Listeria* (L.) *monocytogenes* is of great importance from a food safety perspective, and thus for human health. The consequences of failures in this regard have been exemplified by recent large listeriosis outbreaks in the USA and Europe. It is thus particularly notable that tolerance to quaternary ammonium compounds such as benzalkonium chloride (BC) has been observed in many *L. monocytogenes* strains. However, the molecular determinants and mechanisms of BC tolerance of *L. monocytogenes* are still largely unknown. Here we describe Tn*6188*, a novel transposon in *L. monocytogenes* conferring tolerance to BC. Tn*6188* is related to Tn*554* from *Staphylococcus* (S.) *aureus* and other Tn*554*-like transposons such as Tn*558*, Tn*559* and Tn*5406* found in various *Firmicutes*. Tn*6188* comprises 5117 bp, is integrated chromosomally within the *radC* gene and consists of three transposase genes (*tnpABC*) as well as genes encoding a putative transcriptional regulator and QacH, a small multidrug resistance protein family (SMR) transporter putatively associated with export of BC that shows high amino acid identity to Smr/QacC from *S. aureus* and to EmrE from *Escherichia coli*. We screened 91 *L. monocytogenes* strains for the presence of Tn*6188* by PCR and found Tn*6188* in 10 of the analyzed strains. These isolates were from food and food processing environments and predominantly from serovar 1/2a. *L. monocytogenes* strains harboring Tn*6188* had significantly higher BC minimum inhibitory concentrations (MICs) (28.5 ± 4.7 mg/l) than strains without Tn*6188* (14 ± 3.2 mg/l). Using quantitative reverse transcriptase PCR we could show a significant increase in *qacH* expression in the presence of BC. *QacH* deletion mutants were generated in two *L. monocytogenes* strains and growth analysis revealed that Δ*qacH* strains had lower BC MICs than wildtype strains. In conclusion, our results provide evidence that Tn*6188* is responsible for BC tolerance in various *L. monocytogenes* strains.

## Introduction

*L. monocytogenes* is a gram-positive facultative intracellular food-borne pathogen responsible for listeriosis, a rare but severe illness in humans and also in animals. First unequivocal proof that dairy products can be responsible for listeriosis outbreaks was provided in the early 1980s [1,2]. Since then the importance of *L. monocytogenes* as a food-borne pathogen became more and more obvious. Concomitantly, an increasing rate of listeriosis cases in some European countries has been reported during the last few years [3,4]. In addition, there have been a number of recent large listeriosis outbreaks e.g. in 2011 in the USA [5] and in 2009/2010 in Austria, Germany, and the Czech Republic [6,7].

*L. monocytogenes* can survive in multiple habitats and has been isolated from soil, silage, marine and fresh water, sewage, vegetation, food processing plants, food, domestic and wild animals as well as humans [8,9]. Due to the ability of *L. monocytogenes* to resist environmental stresses, which normally limit bacterial growth and survival such as heavy metal ions, high salt concentration, low pH-values, low temperatures, as well as low water activity, this pathogen successfully colonizes food processing environments. Quaternary ammonium compounds (QACs) such as benzalkonium chloride (BC) or benzethonium chloride are widely used disinfectants in food production [10]. Using disinfectants at recommended concentrations, *L. monocytogenes* can be completely inactivated; however factors such as food debris, biofilm formation, inadequate cleaning and disinfection procedures or dosage failure can significantly reduce the efficiency of disinfectants [11-13]. A regular exposure to sublethal concentration of disinfectants can lead to the development of tolerance [14,15]. Between 10 to 46% of *L. monocytogenes* strains isolated from food and food processing environments can be regarded as being BC tolerant [16-19]. However, the molecular mechanisms underlying the resistance of *L. monocytogenes* to organic sanitizers are still poorly understood. Recently, a plasmid-borne composite transposon responsible for BC tolerance was identified in the *L. monocytogenes* strain (H7550) responsible for an outbreak in 1998/1999 in the USA [20]. In that case tolerance to BC was mediated by a *bcrABC* resistance cassette consisting of *bcrA*, a transcriptional regulator and *bcrBC*, representing small multidrug resistance protein family (SMR) transporters.

In this study we have identified and characterized Tn*6188*, a novel transposon in *L. monocytogenes* that confers tolerance to BC.

## Materials and Methods

### Bacterial strains

*L. monocytogenes* strains used in this study (n=91) are shown in Table S1. The strain set comprised reference and field strains isolated from humans, animals, food and food processing environments representing the 12 major serovars: 1/2a, 1/2b, 1/2c, 3a, 3b, 3c, 4a, 4b, 4c, 4d, 4e, and 7. The strains were not selected based on BC resistant phenotype.

### DNA isolation and genome sequencing

Strains were grown overnight in brain heart infusion broth (BHI, Merck; at 37°C with 125 rpm shaking). DNA was isolated from 2 ml of culture using either the DNeasy Blood and Tissue Kit (Qiagen) or the NucleoSpin Tissue Kit (Macherey-Nagel) according to the instructions of the manufacturer. The genomes of *L. monocytogenes* strains 4423 (a serovar 1/2a isolate from smear, Austria) and 6179 (a serovar 1/2a cheese isolate from Ireland previously shown to be tolerant to benzethonium chloride [21]) were sequenced using Illumina paired-end sequencing technology with a read-length of 100 bp on an Illumina GAII genome analyzer available at the University of Veterinary Medicine in Vienna. Four million reads were used for assembly using SeqManNGen (DNASTAR); assembly resulted in 35 and 32 contigs larger than 500 bp for 4423 and 6179, respectively.

### Sequence analyses

The genomes (and the enclosed sequences of Tn*6188*) of *L. monocytogenes* 6179 and 4423 were analyzed and automatically annotated using the Microbial Genome Analysis and Annotation Platform MaGe [22]. Nucleic acid and amino acid sequences were aligned with MAFFT [23]; alignments were visualized using BOXSHADE (http://www.ch.embnet.org/software/BOX_form.html). Sequence searches for Tn*6188* were performed using BlastP and BlastN against the non-redundant (nr) sequences at NCBI GenBank [24].

### PCR screening for Tn*6188*

In total, 91 *L. monocytogenes* strains of different sources were screened for the presence of Tn*6188* (Table S1). PCR primers targeting the *qacH* gene from Tn*6188* and the flanking *radC* gene, into which Tn*6188* is integrated, were designed based on available Tn*6188* and *L. monocytogenes* genome sequences (Table 1). PCR conditions were as follows: 0.2 pmol/µl of each primer, 2mM MgCl_2_, 1mM dNTP-Mix, 0.625U Platinum Taq DNA polymerase (Life Technologies). PCR cycling conditions were: initial denaturation for 5 min at 95°C; 30 cycles of denaturation at 94°C for 40s, annealing at 56°C for 40s and elongation at 72°C (for *qacH* 25s; for *radC* 165s); final elongation at 72°C for 5 min. Negative controls (no DNA added) and positive controls (genomic DNA from *L. monocytogenes* 6179) were included in all PCR reactions. The presence and size of amplification products was checked with agarose gel electrophoresis using SYBR Safe (Life Technologies) or ethidium bromide (Merck) -staining.

**Table 1:** PCR primers used in this study.

**Primer**	**Sequence (5‘-3‘)**	**Amplicon size (bp)**	**Reference**
qacH fwd	ATG TCA TAT CTA TAT TTA GC	366	This study
qacH rev	TCA CTC TTC ATT AAT TGT AAT AG		This study
radC fwd	CTT GCC AAT GAT AAT ATG ATC	200 (- Tn*6188*) 5310 (+ Tn*6188*)	This study
radC rev	GTG GTC TGA ATG CTC CAT CG		This study
BcF5	GGA GGG TAA TCA TGT CAG	1312	[20]
BcR	GTA TAA TCC GGA TGC TGC CC		[20]
16S rRNA fwd	CCC TTA TGA CCT GGG CTA CA	236	This study
16S rRNA rev	CCT ACC GAC TTC GGG TGT TA		This study
qacH SoeA	ATG **GAA** **TTC** AGC TTA TAT TAA TAC *		This study
qacH SoeB	TGT TAT TCG CCC TCC TGA ATC ATC		This study
qacH SoeC	GAT GAT TCA GG AGG GCG AAT AAC AAA TAA CAA GTC CTT CAA ATA ATA CG *		This study
qacH SoeD	AGG **CTG** **CAG** AAG AAG AGA ACG AAC *		This study
qacH SoeE	GCA TTA ATT AAA GGA ATG GTG GAA G		This study
qacH SoeZ	CTC GTA AAC CAA GTA AAA GTC CCC GT		This study
pKSV7 fwd	ATG TGC TGC AAG GCG ATT A		[27]
pKSV7 rev	CCA GGC TTT ACA CTT TAT G		[27]

* regions highlighted in bold represent restriction sites; underlining indicates an overlap with SoeB primer

### PCR screening for *bcrABC*

All 91 strains were also screened for the presence of the *bcrABC* resistance cassette using primers BcF5 and BcR targeting the *bcrABC* genes [20], (Table 1). PCR conditions were as described above using an annealing temperature of 62°C and elongation for 45s. PCR products obtained were sequenced by LGC Genomics.

### Determination of minimum inhibitory concentrations (MICs)

For MIC determination all strains harboring Tn*6188* (CDL67, CDL78, 6179, K15, N22-2, CDL64, F18, F19, F17, 4423) and 10 strains without Tn*6188* (CDL65, NCTC5348, CZ48, F16, R479a, Clone2, CDL77, CDL66, CDL2, 535) as well as Δ*qacH* strains were used. MIC determinations were performed as previously described [19,20]. Briefly, strains were cultivated on tryptic soy agar (Merck). Single colonies were transferred to 8 ml Mueller-Hinton broth (Oxoid) and incubated overnight (37°C, 125 rpm). The bacterial culture was adjusted to an optical density (OD_600_) of 0.1 (corresponding to 10^6^ bacteria per ml as determined by a colony forming unit [CFU] assay). 5 µl of the cultures were spotted in triplicate on Mueller Hinton agar (1.2% agar) supplemented with 1.2% defibrinated sheep blood (Oxoid) containing the following BC (Sigma-Aldrich) concentrations: 0, 2, 5, 10, 15, 20, 25, and 30 mg/l. Plates were incubated for 48h at 30°C. The MIC was defined as the lowest assessed BC concentration which prevented growth. Experiments were performed in three biological independent replicates.

For MIC determination of the antibiotics erythromycin and tetracycline *L. monocytogenes* 4423 and 6179 wildtype and Δ*qacH* deletion mutants were used. MICs were determined as described above for BC using 0, 1, 2, and 5 mg/l erythromycin or tetracycline (Applichem).

### Assessment of growth at different BC concentrations

To investigate the growth of *L. monocytogenes* in the presence of BC in more detail and to determine the BC concentrations suitable for qRT-PCR (see below), two strains - one with (N22-2) and one without (CDL65) Tn*6188* - were selected (Table S1). One *L. monocytogenes* colony was grown overnight in 8 ml BHI (37°C, 125 rpm); the bacterial culture was adjusted to an optical density (OD_600_) of 0.2 with minimal media (MM) consisting of RPMI-1640 supplemented with 1% L-glutamine (both PAA) and 0.08 mg/ml ferric citrate (Merck); and incubated in 10 ml MM using 100 ml flasks (at 37°C; 125 rpm) with the following BC concentrations: 0, 1, 2, 5 mg/l; OD_600_ was measured at time point 0, 2, 4, 6, 8, 10 and 24h and mean values and SD of at least two biological independent replicates were calculated.

### RNA isolation, transcription into cDNA

To investigate the expression of the *qacH* gene, one colony of *L. monocytogenes* 4423 was grown overnight in 8 ml BHI broth at 37°C with shaking (125 rpm). The bacterial culture was adjusted to an optical density (OD_600_) of 0.1 in a final volume of 65 ml BHI broth. Cells were grown at 37°C with shaking (125 rpm) to an OD_600_ of 0.5. Cultures were split in four parts of 15 ml each and incubated with BC (Sigma-Aldrich) at the following BC concentrations: 0, 1, 2, 5 mg/l for 30 min at 37°C with shaking. Cells were harvested by centrifugation (4000 rpm, 5 min.) and washed with 5 ml of PBS (phosphate buffered saline). Pellets were resuspended in 1 ml RNAlater Solution (Life Technologies) and stored at 4°C for 48h until RNA isolation. Experiments were performed in three biological independent replicates.

For RNA isolation, pellets were resuspended in 750 µl TRIzol Reagent (Life Technologies). Cells were disrupted using beadbeating in Lysing Matrix A tubes (MP Biomedicals) with a FastPrep FP120 instrument (MP Biomedicals) with the following parameters: three times 45s at speed 5.5 at 4°C. RNA isolation was performed according to the manufacturer’s instructions. Concentration of nucleic acids was measured with a Nanodrop instrument (Shimadzu BioSpec-nano) and remaining DNA was removed using the Turbo DNA-free Kit (Life Technologies) according to manufacturer’s instructions. The absence of DNA was confirmed by performing a PCR using qacH fwd and rev primers. PCR conditions were the same as indicated above, except that 40 cycles were used.

200 ng RNA were used for cDNA synthesis with random primers using the RevertAid H Minus First Strand cDNA Synthesis Kit (Thermo Scientific) according to the protocol of the manufacturer.

### Quantitative real time (qRT)-PCR

Primers targeting the Tn*6188 qacH* and *L. monocytogenes* 16S rRNA gene were designed using the online tools Primer3 (v. 0.4.0), Primer Express 2.0 and NetPrimer (Premier Biosoft) (Table 1). We used the 16S rRNA, which is the most stably expressed housekeeping gene of *L. monocytogenes* under various growth conditions [25], as an internal reference. 0.5 ng cDNA were used as a template in each reaction. Cycling conditions for the *qacH* qRT-PCR consisted of: 94°C for 2 min., 40 cycles consisting of the two steps 94°C for 30s and 60°C for 1 min., and a subsequent dissociation curve (95°C for 1 min., 50-95°C for 30s each) using a Mx3000P cycler (Stratagene). In a final volume of 25 µl following concentrations were applied: 0.25 pmol/µl primers, 3.5 mM MgCl_2_, 0.8 mM dNTP-Mix, 1.5 U Platinum Taq DNA polymerase (Life Technologies) and 1x EvaGreen (Jena Bioscience). The same concentrations were also used for the 16S rRNA gene qRT-PCR except for 2 mM MgCl_2_. Cycling conditions for the 16S rRNA gene qRT-PCR consisted of: 95°C for 4 min., 40 cycles of the three steps: 95°C for 10s and 70°C for 20s and 72°C for 20s. A subsequent dissociation curve (95°C for 1 min., 50-95°C for 30s each) was performed. A dilution series of genomic DNA from *L. monocytogenes* 4423 (1 to 10^-5^ ng/µl) was used as an internal amplification control and for calculation of the primer efficiency (1.84 for *qacH* and 1.7 for 16S rRNA). Data were analyzed using Mx300P MxPro software (Stratagene). Each sample was measured in duplicates. Relative quantification was performed using the comparative Ct method. Values, given as x-fold of control (0 mg/l BC) were normalized using 16S rRNA.

### Creation of *qacH* deletion mutants

Non-polar deletion of the *qacH* gene was performed in two *L. monocytogenes* strains (4423 and 6179) using the splicing overlap extension PCR technique (SOEing-PCR) [26] and the temperature-sensitive shuttle vector pKSV7 [27], conferring both ampicillin and chloramphenicol resistance. Primers (SoeA-D) were designed to amplify two segments of DNA (SoeAB and SoeCD), which flank the region that is targeted for mutagenesis (Table 1). PCR reactions were performed using VENT^®^ DNA Polymerase (New England BioLabs). The amplicons were purified with the QIAquick PCR purification kit (QIAGEN), mixed in a 1:1 ratio and used as a template for another PCR using primer SoeA and SoeD to create a spliced amplicon (SoeAD). This PCR product was gel extracted (QIAquick gel extraction kit, Qiagen), digested with EcoRI and PstI (Roche) and ligated using T4 ligase (Roche) into a similarly digested pKSV7, resulting in pKSV7(SoeAD). pKSV7(SoeAD) was transformed into *E. coli* using One Shot TOP10 chemically competent *E. coli* (Life Technologies), transformants were selected on TSA agar complemented with 50 µg/ml ampicillin (Sigma Aldrich), the plasmid was isolated using QIAprep spin miniprep kit (QIAGEN) and electroporated into two competent *L. monocytogenes* strains (4423 and 6179) with the following parameters: voltage 2 kV, resistance 400 Ω, capacity 25 µF. Transformants were selected on BHI agar complemented with 10 µg/ml chloramphenicol (BHI-Cm, Sigma-Aldrich). Chromosomal integration of the plasmid was induced by serial passage of transformants in BHI-Cm at 42°C. Plasmid excision and curing was induced by continuous passage in BHI at 30°C. Every 4^th^ to 5^th^ passage cultures were plated on BHI agar and colonies were streaked in replica on BHI and BHI-Cm agar to select the appropriate deletion event. Chloramphenicol sensitive strains, i.e. a phenotype indicating the loss of the pKSV7 plasmid, were selected for further analysis. Deletion mutants were identified by PCR primers designed to bind upstream of SoeA (SoeZ) and downstream of SoeD (SoeE). These yielded either a 799 bp (mutant) or 1171 bp (wildtype) amplicon. In addition, a PCR amplifying the *qacH* gene was also performed on wildtype and mutant strains. The in-frame gene deletion in the mutant strain was confirmed by sequencing the SoeEZ PCR product (LGC Genomics).

### Statistical analysis

Averages and standard deviations were calculated using Microsoft Excel^®^ 2007 software. Values were compared statistically using t-test (independent variables); P-values <0.05 were considered to be significant.

### Accession numbers

The nucleotide sequences of Tn*6188* from *L. monocytogenes* 6179, 4423, and K15 have been submitted to EMBL/DDBJ/GenBank under accession numbers HF565366, HF565365, and HG329628, respectively.

## Results and Discussion

**Figure 1:**
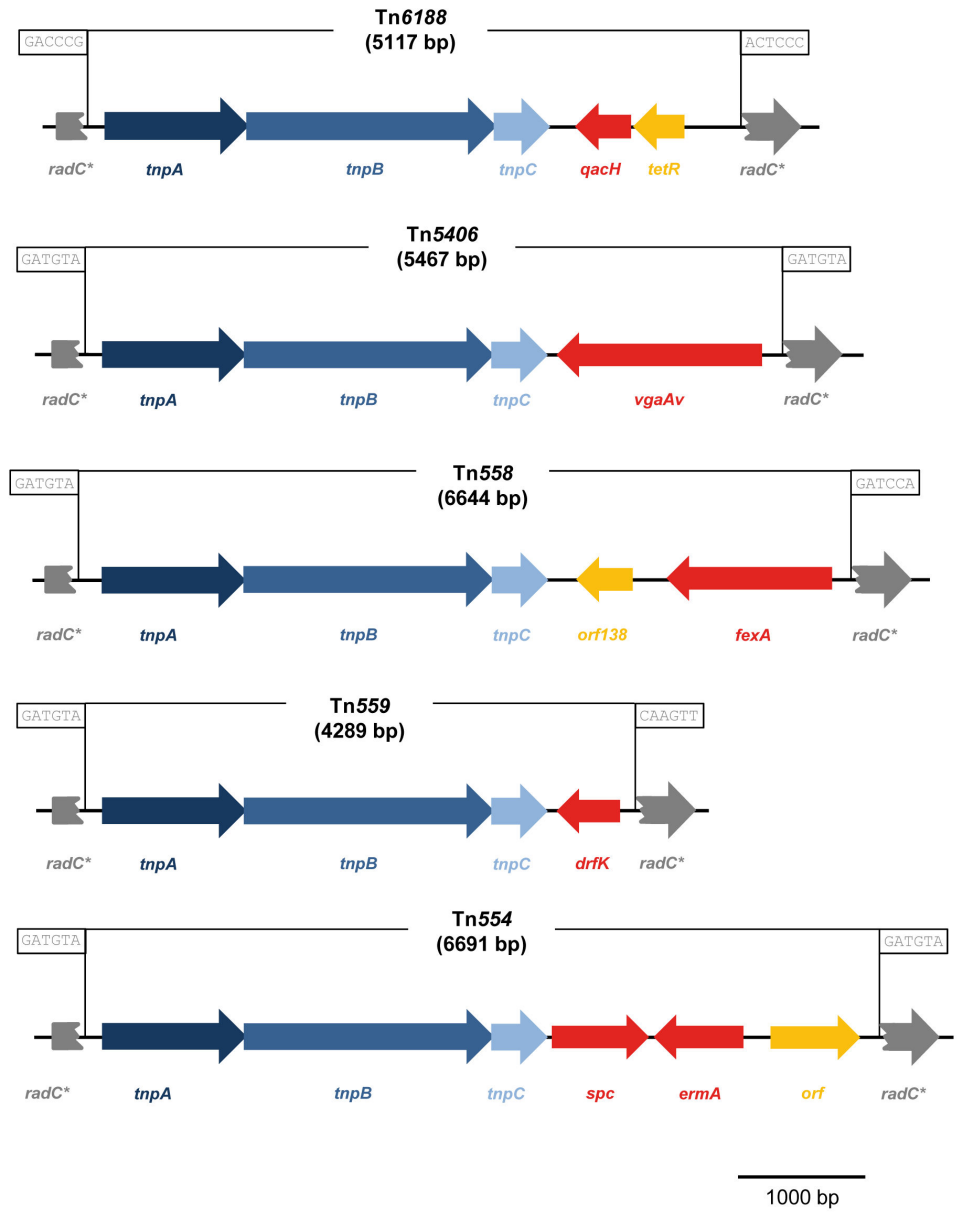
Organization of the *L. monocytogenes* transposon Tn*6188* and comparison with other related transposons Tn*554* (X03216), Tn*5406* (AF186237), Tn*558* (AJ715531), Tn*559* (FN677369). The transposase genes *tnpABC* are shown in dark blue, blue and light blue, respectively. Effector proteins conferring antimicrobial resistance are shown in red; proteins with other or unknown function are shown in orange. The 6 bp nucleotide sequences at the transposon junctions are shown in boxes. The disrupted *radC* genes are indicated by an asterisk.

**Figure 2:**
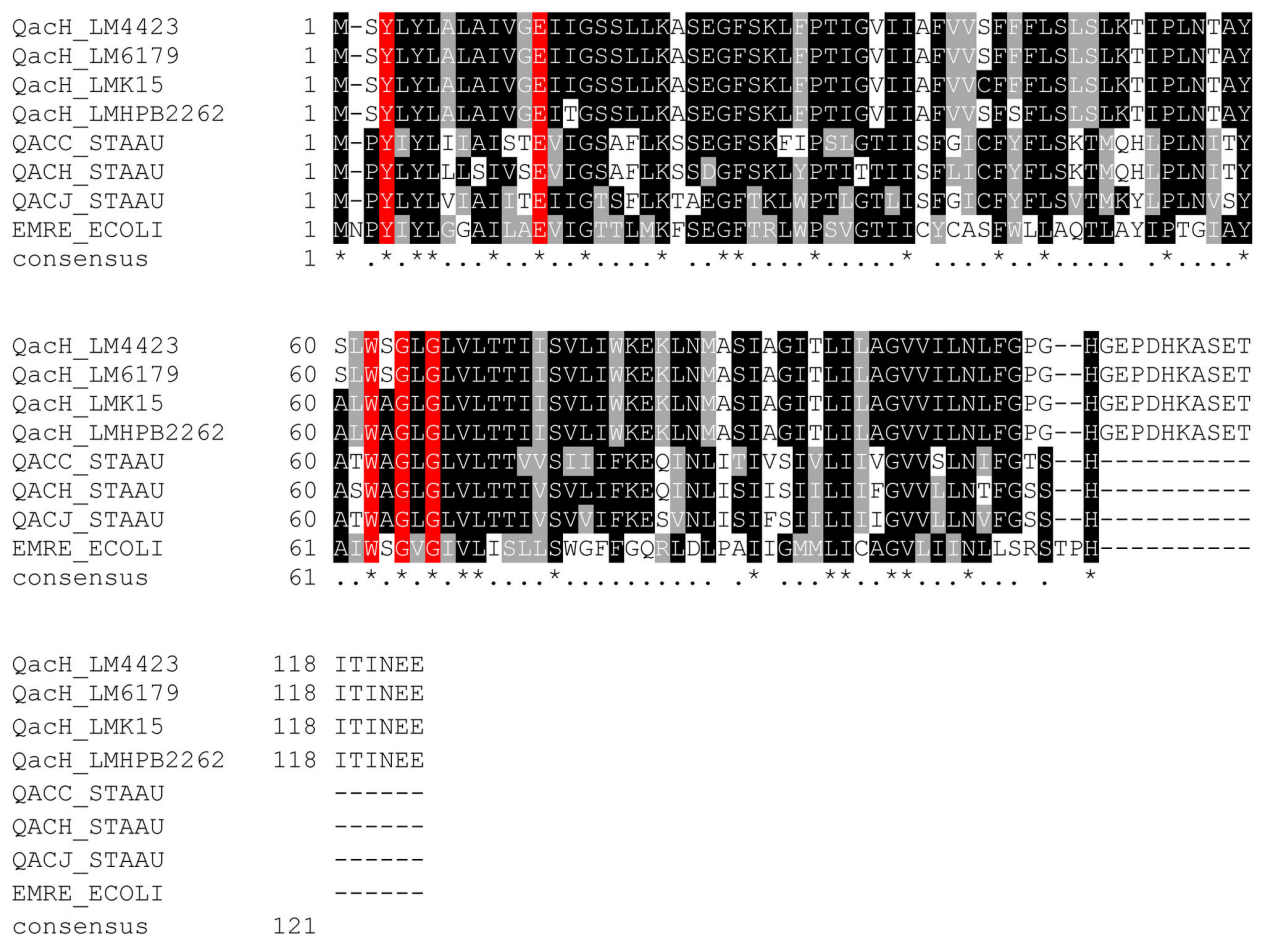
Amino acid sequence alignment of QacH from Tn*6188* and related Qac/smr/EmrE-like proteins. The alignment was done with MAFFT [23], shading of conserved amino acid residues was performed with Boxshade. Amino acid residues important for function in EmrE and QacC are highlighted in red. Data for important amino acid residues are taken from [31-33]. The consensus is displayed at the bottom of each alignment block, asterisks indicate identical positions, dots indicate similar positions. Abbreviations and accession numbers: QacH_LM4423 (*L. monocytogenes* 4423, CCP37730), QacH_LM6179 (*L. monocytogenes* 6179, CCP37735), QacH_LMK15 (*L. monocytogenes* K15, HG329628), QacH_LMHPB2262 (*L. monocytogenes* HPB2262, EFF95069), QACC_STAAU (*Staphylococcus aureus*, P14319), QACH_STASA (*Staphylococcus saprophyticus*, O87868), QACJ_STAAU (*Staphylococcus aureus*, NP_783299), EMRE_ECOLI (*E. coli*, P23895).

During genome analysis of the persistent *L. monocytogenes* strains 6179 and 4423 we identified a region in both genomes harboring three consecutive transposase genes showing high similarity to the transposases of Tn*554* and related transposons from *S. aureus* and other *Firmicutes*. After a more detailed inspection we identified a 5117 bp region, which is not flanked by inverted repeats, representing a novel transposon named Tn*6188*. Tn*6188* has a G+C content of 32.9%, which is considerably lower than the average G+C content of *L. monocytogenes* 4423 and 6179 (37.9%, respectively) and other *L. monocytogenes* genomes (37.8 to 38.0%). The Tn*6188* copies from *L. monocytogenes* strains 6179 and 4423 are identical. Tn*6188* encodes three consecutive transposase genes (*tnpABC*), one protein with high similarity to Smr/EmrE/Qac proteins, i.e. transporters responsible for the export of disinfectants from *E. coli* and *S. aureus* [28], and a putative tetR family transcriptional regulator upstream of the transporter (Figure 1, Table 2). The Smr/EmrE/Qac-like protein consists of 123 amino acids and shares highest amino acid sequence identity, among functionally characterized proteins, with QacH from *Staphylococcus saprophyticus* (59%) [29], QacJ from *S. aureus* (57%) [30], QacC/Smr from *S. aureus* (53%) [31,32], and with EmrE from *E. coli* (45%) [33,34] (Figure 2). We thus refer to this protein as QacH. These transporters belong to the small multidrug resistance (SMR) protein family (Transporter Classification Database family: 2.A.7.1 [35]), comprise approx. 100 to 140 amino acids, span the membrane by four transmembrane alpha helices and confer tolerance to a variety of QACs such as BC or cetyltrimethylammonium bromide (CTAB) [28]. Functionally characterized members of the SMR family catalyze multidrug/H^+^ antiport with the proton motive force providing the driving force for drug efflux [28,33]. Amino acids important for the functionality of SMR family transporters are also conserved in QacH of Tn*6188*. Interestingly, QacH from Tn*6188* shows a 16 amino acid C-terminal extension compared to functionally characterized Smr/Qac/EmrE proteins, which might result in functional differences of these transporters (Figure 2).

**Table 2:** Predicted genes on Tn*6188*.

**Gene**	**function**	**start**	**end**	**length (amino acids)**	**G+C-content**	**Best blast hit (BlastP nr) (amino acid identity to best blast hit excluding *Listeria*, GenBank accession no. of best hit)**
*tnpA*	Transposase	132	1217	1086 bp (361)	33.0%	Transposase A Tn*558 Staphylococcus lentus* (91%, CAG29647)
*tnpB*	Transposase	1220	3133	1914 bp (637)	33.7%	Transposase B Tn*558 Staphylococcus lentus* (93%, CAG29648)
*tnpC*	Transposase	3135	3500	366 bp (121)	36.1%	Transposase C Tn*558 Staphylococcus lentus* (88%, CAG29649)
*qacH*	Quaternary ammonium compound-resistance protein QacH	3765	4135	372 bp (123)	33.6%	DMT superfamily multidrug transporter *Sporosarcina newyorkensis* 2681 (89%, ZP_08677172)
*tetR*	Transcriptional regulator, tetR family	4138	4539	519 bp (172)	32.2%	TetR family transcriptional regulator *Sporosarcina newyorkensis* 2681 (77%, ZP_08677171)

Notably, Tn*554* and related transposons (Tn*558*, Tn*559*, Tn*5406*) show a strong preference for insertion at the same relative (highly conserved) site within the *radC* gene found in many *Firmicutes* [36-41]. Similarly, Tn*6188* is inserted into the same relative site within the *radC* gene between bp 540 and 541. This conservation of the insertion site is surprising as the RadC proteins from *L. monocytogenes* show only 45% amino acid sequence identity to the RadC proteins from *Staphylococcus* spp. and 53% to 60% to *Bacillus* spp. We performed amino acid and nucleic acid alignments of radC sequences form various *Listeria* spp. and other *Firmicutes*. The amino acid alignment shows a highly conserved motif upstream of the insertion site (Figure S1). Also on DNA level, a highly conserved region upstream of the insertion site of Tn*6188* and other Tn*554*-like transposons is present (Figure S2). We also performed BlastP searches against all *Listeria* genomes in GenBank using the *L. monocytogenes* EGDe RadC amino acid sequence as query and found RadC proteins in all *Listeria* species with sequenced genomes. The *Listeria* homolog with the lowest amino acid identity was found in *L. grayi* (64%). Many different functions of RadC proteins e.g. DNA repair, a role in transformation or competence, have been suggested, however, the precise function of RadC is still unclear [42]. Tn*6188* positive strains can be considered to be *radC* insertion mutants; however as the function of RadC is still unknown, it is hard to determine the effect of the *radC* insertion. In *Bacillus subtilis* and *Streptococcus pneumoniae radC* is non-essential [42]. The transposase genes required for transposition of Tn*554*-like transposons are highly conserved, but the resistance markers are diverse. Tn*554*-like transposons are thus flexible units allowing the transfer of a variety of resistance markers among *Firmicutes*. Tn*6188* is the first Tn*554*-like transposon which encodes a SMR family transporter as a resistance marker and provides tolerance to disinfectants rather than the resistance to antibiotics provided by Tn*554*, Tn*558*, Tn*559* and Tn*5406*. Tn*554*-like transposons thus share a modular composition with a highly conserved transposase module (consisting of the transposases TnpABC) and flexible resistance markers. In general, *Listeria* genomes are believed to be relatively stable and horizontal gene transfer (particularly from outside the genus *Listeria*) is thought to be relatively uncommon [43]. The overall G+C content of Tn*6188* (32.9%), being considerably lower than the overall G+C content of *Listeria* genomes, suggests a relatively recent transfer of Tn*6188* into *L. monocytogenes*. In line with this, to our knowledge, only few transposons have been identified in *L. monocytogenes* so far: Tn*5422*, which is found on the majority of *Listeria* plasmids, conferring tolerance to cadmium [44-46], the IS*1216* composite transposon harboring the *bcrABC* resistance cassette [20], Tn*6198*, a conjugative Tn*916*-like transposon conferring resistance to antibiotics [47], a Tn*554*-like transposon putatively involved in arsenic resistance [48], and an integrative conjugative element belonging to the Tn*916* family which has been found in the *L. monocytogenes* EGDe genome [49]. In contrast to Tn*6188*, Tn*5422* and Tn*6198* have a G+C content of 37.9%, which is much more similar to the overall G+C content of *L. monocytogenes* genomes. This suggests that the transfer of Tn*5422* and Tn*6198* into *L. monocytogenes* was a more ancient event than the introduction of Tn*6188*.

**Figure 3:**
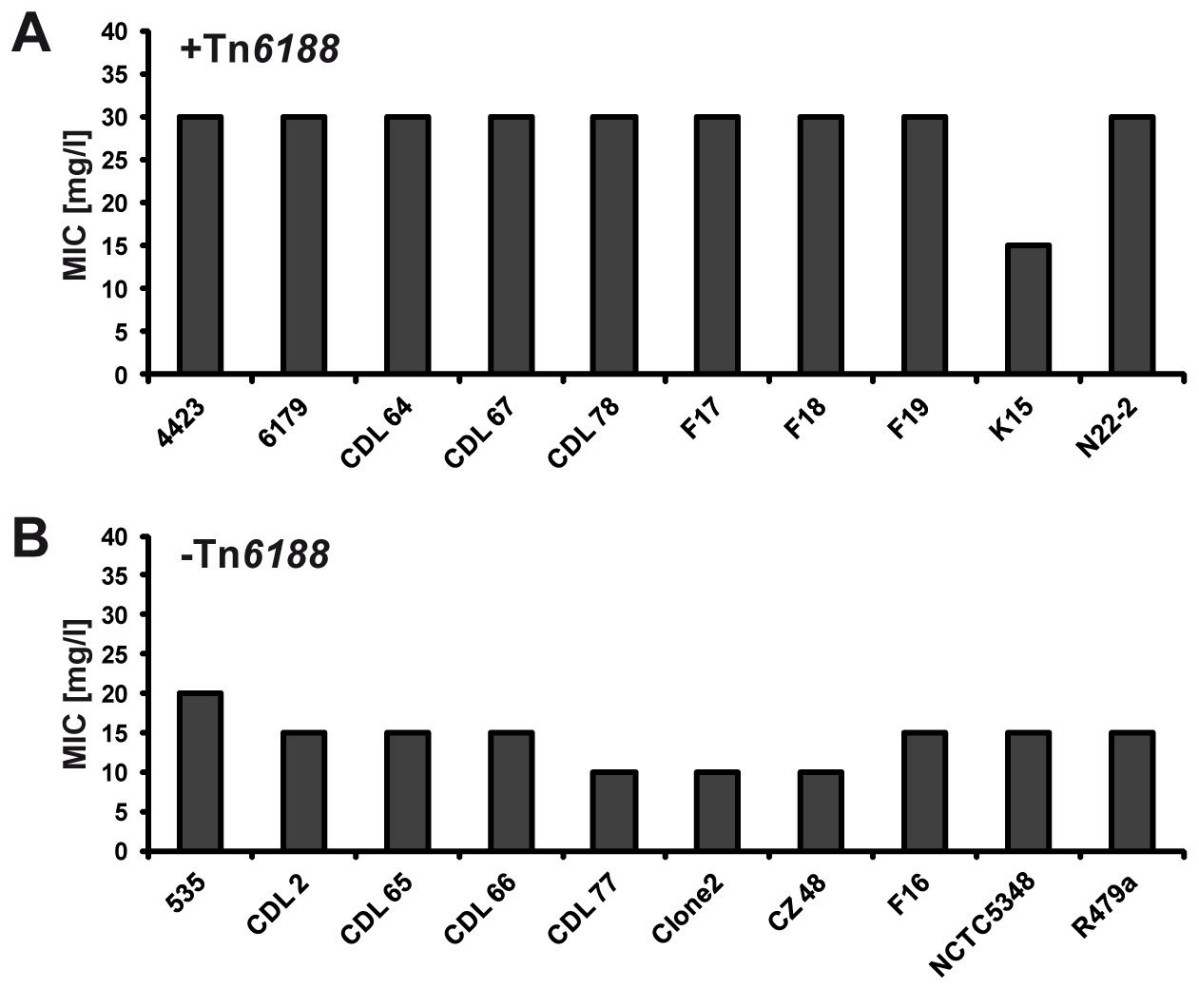
Benzalkonium chloride MICs of *L. monocytogenes* strains. BC MICs of ten *L. monocytogenes* strains with (+Tn*6188*, A) and without (-Tn*6188*, B) Tn*6188* are shown. MICs represent mean values of three biological independent replicates, SD=0.

To investigate the distribution of Tn*6188* across other *L. monocytogenes* strains we first performed BlastP and BlastN searches of the protein and nucleotide sequences against the non-redundant sequences (nr) databases in GenBank. Interestingly, we found only one Tn*6188* copy among all currently sequenced genomes (as of April 2013): The genome of the *L. monocytogenes* strain HPB2262 encodes a Tn*6188* copy sharing 99% nucleotide similarity. Here, Tn*6188* is integrated at the same genomic locus i.e. within the *radC* gene. *L. monocytogenes* HPB2262 (formerly also referred to as Aureli 1997) is a serotype 4b strain which was isolated from a febrile gastrointestinal illness outbreak in Northern Italy in 1997 [50]. The QacH protein of *L. monocytogenes* HPB2262 shares 97% amino acid identity (corresponding to four amino acid substitutions) with QacH from strains 6179 and 4423 (Figure 2). In order to get deeper insights into the prevalence of Tn*6188* among *L. monocytogenes* we designed primers targeting the flanking *radC* and the *qacH* gene of Tn*6188* and performed PCR assays. We screened 91 strains of different serovars isolated from various sources for the presence of Tn*6188* and detected 10 *L. monocytogenes* strains harboring Tn*6188* (11%) (Table S1). As we used primers targeting the flanking *radC* gene, this strongly suggests that in the positive strains Tn*6188* is integrated in the same *radC* locus. Tn*6188* was predominantly found in *L. monocytogenes* 1/2a strains isolated from food and food processing environments. Furthermore, two isolates from smoked salmon (serovar 3a) and cheese processing environment (1/2c) were also found to be positive for Tn*6188* (Table S1). Additionally, we screened all 91 strains for presence of the BC-resistance cassette *bcrABC* [20]. *BcrABC* could be found in 5 of the 91 strains (5.5%), none of them harboring Tn*6188* (Table S1). In order to analyze whether Tn*6188* is involved in BC tolerance we determined the BC MICs of *L. monocytogenes* strains with and without Tn*6188* (n=10, respectively). Strains harboring Tn*6188* showed significantly higher BC MICs compared to strains devoid of Tn*6188* (Figure 3): (28.5 ± 4.7) versus (14 ± 3.2) mg/l, respectively (p < 0.001). Interestingly, strain K15 – although harboring Tn*6188* - showed a BC MIC of only 15 mg/l (Figure 3). In order to get more insights into possible reasons for this reduced MIC we determined the sequence of the Tn*6188* copy in strain K15: it shows 99.9% nucleotide sequence similarity to Tn*6188* from strains 4423 and 6179. Three non-synonymous substitutions were found, all of them in the *qacH* gene, however, the substitutions occur in residues which are not essential for function in other (experimentally characterized) homologous transporters (Figure 2). These results suggest that Tn*6188* in strain K15 is functional and the reduced MIC is likely due to another - yet unknown – reason. Further growth experiments performed in order to identify optimal growth conditions for qRT-PCR, revealed that *L. monocytogenes* without Tn*6188* displayed reduced growth at BC concentrations as low as 1 mg/l whereas strains harboring Tn*6188* showed reduced growth at 2 mg/l BC (Figure S3).

**Figure 4:**
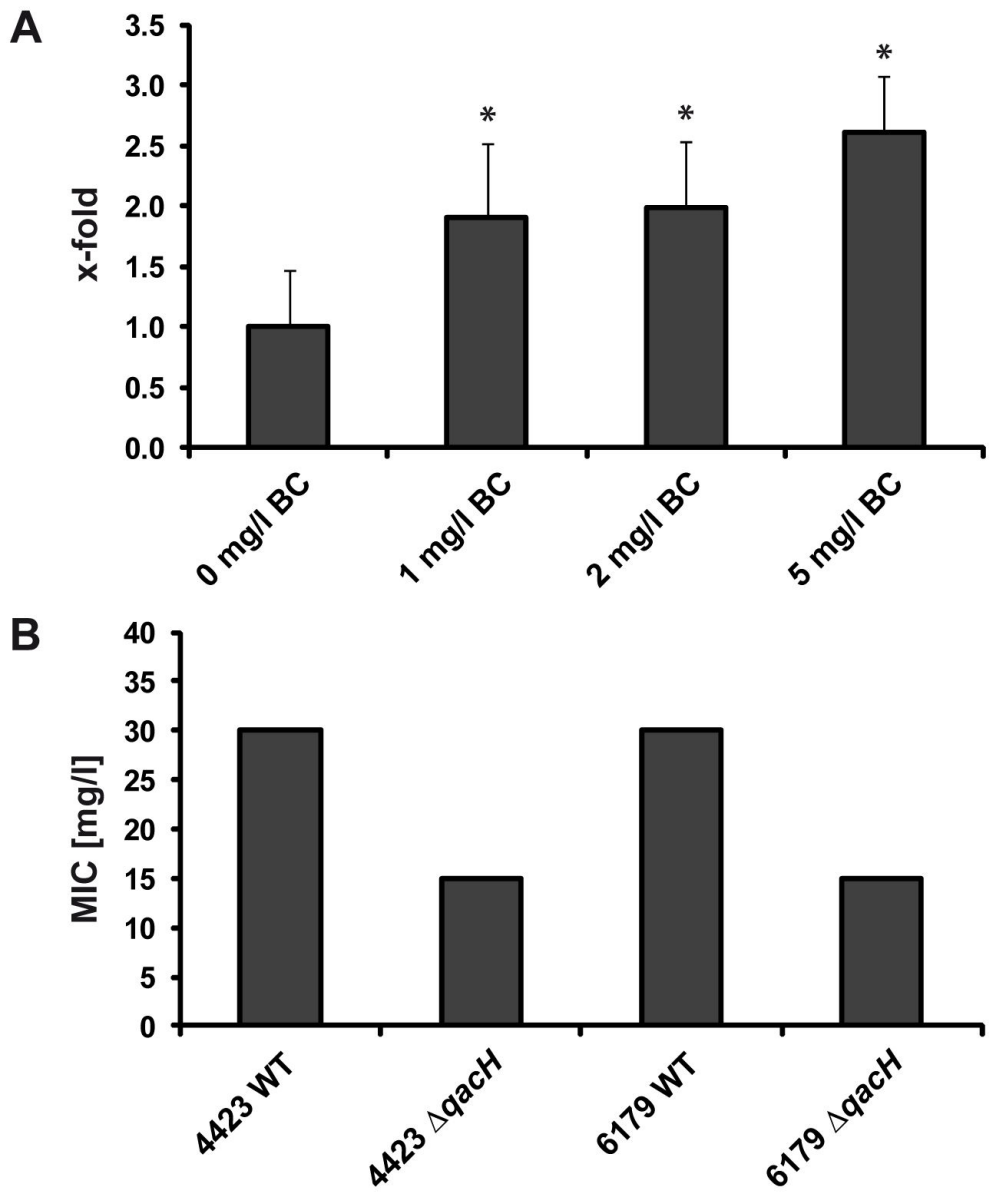
Gene expression of *qacH* and benzalkonium chloride MICs of Δ*qacH* strains. Gene expression of Tn*6188* *qacH* in the presence of different BC concentrations was determined by quantitative RT-PCR (**A**). Values, represented as x-fold of control (0 mg/l BC), were normalized according 16S rRNA expression levels. *p<0.02. Differences in MICs of BC using *L. monocytogenes* wildtype and Δ*qacH* strains (4423 and 6179) are shown in (**B**). MICs represent mean values of three biological independent replicates, SD=0.

To get a better insight into whether Tn*6188* is involved in BC resistance, we investigated the expression of the *qacH* gene in the presence of BC by quantitative RT-PCR: mRNA expression of *qacH* was significantly (p<0.02) higher (between 1.9 and 2.6 fold) in the presence of BC (Figure 4A).

In order to provide additional evidence of the role of Tn*6188* in BC resistance we generated non-polar deletion mutants of the *qacH* gene in two *L. monocytogenes* strains (4423 and 6179, Figure S4). We subsequently determined the BC MICs comparing the Δ*qacH* with the wildtype strains: both Δ*qacH* strains showed a significantly lower BC MIC than the wildtype strains (15 mg/l compared to 30 mg/l, p< 0.0001, Figure 4B). The MICs observed here are comparable to the BC MICs determined for the *bcrABC* resistance cassette: 40 mg/l for the strains harboring the *bcrACB* cassette and 10 mg/l for strains without the resistance cassette [20]. In addition, growth experiments revealed no differences in the growth of wildtype and mutant strains without BC, whereas reduced growth of the Δ*qacH* strains at concentrations as low as 1mg/l BC was observed (Figure S5). However, definite proof of the role of Tn*6188* in BC resistance would require complementation of the mutants.

EmrE has been described to provide also resistance to erythromycin and tetracycline [34], however another more recent study could not show an involvement of EmrE in resistance to erythromycin or other antibiotics [51]. We therefore tested whether erythromycin and tetracycline might be possible substrates for QacH and determined MICs for these antibiotics using *L. monocytogenes* 4423 and 6179 wildtype and Δ*qacH* strains. MICs were 1 mg/l for erythromycin and 2 mg/l for tetracycline for both wildtype and Δ*qacH* strains (data not shown). These results suggest that Tn*6188* is not involved in resistance to erythromycin or tetracycline.

Tolerance to BC has been observed among *L. monocytogenes* strains in many previous studies [16-19,52-55]. However, with one exception, the molecular mechanisms and genetic markers conferring BC tolerance in *L. monocytogenes* are still largely unknown. The only characterized BC tolerance mechanism in *L. monocytogenes* is an IS*1216* composite transposon on plasmid pLM80 of the strain H7550 harboring (among others) a *bcrABC* resistance cassette consisting of two SMR family transporters and a tetR family transcriptional regulator [20]. The transporters BcrBC show 50% and 53% amino acid sequence identity to QacH from Tn*6188*, the transcriptional regulator BcrA shows 38% amino acid identity to TetR from Tn*6188*. Apart from the similarity between the transporters and the regulator, no similarities between the IS*1216* composite transposon and Tn*6188* are found. Despite the differences between these two transposon-associated elements with respect to transposases and location (chromosome versus plasmid), both encode mechanisms for transposon-based disinfectant resistance. However, without detailed knowledge of the distribution and dissemination mechanisms of both elements, it is difficult to compare their distribution.

Taken together, several lines of evidence strongly suggest a role of Tn*6188* in BC resistance: (i) The BC MICs of *L. monocytogenes* strains harboring Tn*6188* are significantly higher than those of strains without Tn*6188* (ii). The expression of *qacH* is significantly higher in the presence of BC and (iii) Δ*qacH* strains show reduced BC MICs. The BC MICs found in this study (10 to 30 mg/l) are in the same range as those described in other studies [17,19,20]. It is, however, important to keep in mind that the usage of appropriate sanitizer concentrations will efficiently eradicate *L. monocytogenes*. However, the higher BC resistance of Tn*6188* positive strains might nevertheless be an important advantage in food processing environments if e.g. dosage failures during sanitation occur or in niches which are difficult to sanitize e.g. where biofilms can form. In such niches the effective concentrations of sanitizers might be significantly lower and Tn*6188* positive strains might better survive.

The identification and characterization of Tn*6188* provides evidence for a novel transposon-based molecular mechanism for BC resistance and sheds new light on horizontal gene transfer in *L. monocytogenes*.

## Supporting Information

Table S1 ***Listeria monocytogenes* strains used and results of PCR screening for Tn*6188* and *bcrABC*.**
(PDF)

Figure S1 **Amino acid based alignment of RadC proteins from various *Firmicutes*.**
(PDF)

Figure S2 **Nucleic acid based alignment of *radC* sequences from various *Firmicutes*.**
(PDF)

Figure S3 **Growth of two *L. monocytogenes* strains with and without Tn*6188* in the presence of different BC concentrations (0-5 mg/l) at 37°C.**
(PDF)

Figure S4 **PCR analysis of *L. monocytogenes qacH* deletion mutants.**
(PDF)

Figure S5 **Growth of *L. monocytogenes* 4423 wildtype strain and *L. monocytogenes* 4423 Δ*qacH* strain in the presence of different BC concentrations (0-5 mg/l) at 37°C.**
(PDF)
