# Supplementary material for: Tn6188 - A Novel Transposon in Listeria monocytogenes Responsible for Tolerance to Benzalkonium Chloride
Source: PLoS One. 2013 Oct 2;8(10):e76835. doi: 10.1371/journal.pone.0076835 (PMC3788773; doi:10.1371/journal.pone.0076835)
Supplement: Table S1 — Listeria monocytogenes strains used and results of PCR screening for Tn6188 and bcrABC. (PDF) [file pone.0076835.s001.pdf]

Table S1: *Listeria monocytogenes* strains used and results of PCR screening for Tn*6188* and *bcrABC*

Code		Source	Serovar	*radC* PCR	*qacH* PCR	*bcrABC* PCR
NCTC 5105*	Reference strains	unknown	3a	-	-	-
CIP 78.36**			3c	-	-	-
NCTC 10527*			4b	-	-	-
CIP 58.56**			4e	-	-	-
CIP 60.91**			4e	-	-	-
**CDL 2**	**animal**	**sheep, abortus**	**1/2a**	-	-	-
NCTC 10357*		rabbit	1/2a	-	-	-
NCTC 10887*		chinchilla	1/2b	-	-	-
CIP 105458**		sheep	4d	-	-	-
ATCC 19118***		chicken	4e	-	-	-
CDL 129	human	cerebrospinal fluid	1/2a	-	-	-
CDL 130		blood	1/2a	-	-	-
CDL 178		blood	1/2a	-	-	-
Clone 1		blood	1/2a	-	-	-
**Clone 2**		**blood**	**1/2a**	**-**	**-**	**-**
CDL 131		blood	1/2b	-	-	-
CDL 132		blood	1/2b	-	-	-
CDL 133		blood	1/2b	-	-	-
**NCTC 5348***		**cerebrospinal fluid**	**1/2c**	-	-	-
CDL 182		blood	1/2c	-	-	+
CIP 80.10**		infant	3b	-	-	-
CIP 78.35**		spinal fluid	3b	-	-	-
NCTC 4885*		meningitis	4b	-	-	-
CDL 123		blood	4b	-	-	-
CDL 124		blood	4b	-	-	-
CDL 127		blood	4b	-	-	-
CDL 136		peritoneal	4b	-	-	-
CDL 137		blood	4b	-	-	-
CDL 185		blood	4b	-	-	-
CDL 186		blood	4b	-	-	-
CIP 101821**		urine	4b	-	-	-
CIP 78.43**		feces	7	-	-	-
CZ 70	food	blue veined cheese	1/2a	-	-	-
CDL 69		smoked salmon	1/2a	-	-	+
**F 16**		**smoked salmon**	**1/2a**	-	-	-
**F 17**		**smoked salmon**	**1/2a**	**+**	**+**	-
**F 18**		**smoked trout**	**1/2a**	**+**	**+**	-
**F 19**		**smoked trout**	**1/2a**	**+**	**+**	-
**R479a**		**smoked salmon**	**1/2a**	-	-	-
**CDL 64**		**ham**	**1/2a**	**+**	**+**	-
**CDL 65#**		**minced chicken**	**1/2a**	-	-	-
**CDL 66**		**minced beef**	**1/2a**	-	-	-
**CDL 67**		**salami**	**1/2a**	**+**	**+**	-
CDL 174		cheese rind	1/2a	-	-	-
**6179**		**cheese**	**1/2a**	**+**	**+**	-
CDL 175		cheese	1/2a	-	-	-
CDL 154		cheese	1/2a	-	-	-
CDL 68		minced pork	1/2a	-	-	-
CDL 150		cheese rind	1/2a	-	-	-
3261		cheese	1/2a	-	-	-
CDL 71		tuna fish	1/2b	-	-	-
251		cheese rind	1/2b	-	-	-
CDL 147		cheese	1/2b	-	-	-

CDL 143		cheese	1/2b	-	-	-
CDL 161		cheese	1/2b	-	-	-
CDL 72		minced pork	1/2b	-	-	+
CDL 73		minced chicken	1/2c	-	-	-
CDL 75		salami	1/2c	-	-	-
CIP 103573**		milk	1/2c	-	-	-
CDL 170		cheese	1/2c	-	-	-
CDL 172		cheese	1/2c	-	-	-
CDL 76		smoked salmon	1/2c	-	-	+
**CDL 77**		**minced chicken**	**3a**	**-**	**-**	**-**
**CDL 78**		**smoked salmon**	**3a**	**+**	**+**	**-**
CDL 157		cheese	4b	-	-	-
CDL 158		cheese	4b	-	-	-
CDL 70		salami	4b	-	-	-
CDL 164		cheese	4b	-	-	-
**535**		**cheese**	**4b**	**-**	**-**	**-**
CDL 176		cheese	4b	-	-	-
CDL 79		salami	4b	-	-	-
CDL 80		salami	4b	-	-	-
3243	Food processing environment	smear water	1/2b / 3b	-	-	-
3262		smear water	1/2a	-	-	-
**4423**		**smear**	**1/2a**	**+**	**+**	**-**
4898		washing water	1/2a	-	-	-
CDL 153		smear	1/2a	-	-	-
**CZ 48**		**swab**	**1/2a**	**-**	**-**	**+**
**N 22-2#**		**floor**	**1/2a**	**+**	**+**	**-**
W 33		swab	1/2a / 3a	-	-	-
W 34		swab	1/2a / 3a	-	-	-
W 35		swab	1/2a / 3a	-	-	-
W 36		swab	1/2a / 3a	-	-	-
CDL 9		washing water	1/2b	-	-	-
CDL 142		drain water	1/2b	-	-	-
CDL 146		brine	1/2b	-	-	-
CDL 162		brine	1/2b	-	-	-
CDL 171		smear	1/2c	-	-	-
**K15**		**drain water**	**1/2c / 3c**	**+**	**+**	**-**
CDL 177		drain water	4b	-	-	-
3202		smear water	1/2b / 3b	-	-	-

Strain Collection of Institute for Milk Hygiene, University of Veterinary Medicine Vienna, Austria;
*NCTC National Collection of Type Cultures, London, UK;
**CIP Biological Resource Centre of the Pasteur Institute, Paris, France;
***ATCC American Type Culture Collection, Wesel, Germany;
Strains used for MIC determination are shown in boldface letters and highlighted in grey;
Strains used for growth curves
