# Supplementary material for: Tn6188 - A Novel Transposon in Listeria monocytogenes Responsible for Tolerance to Benzalkonium Chloride
Source: PLoS One. 2013 Oct 2;8(10):e76835. doi: 10.1371/journal.pone.0076835 (PMC3788773; doi:10.1371/journal.pone.0076835)
Supplement: Figure S1 — Amino acid based alignment of RadC proteins from various Firmicutes. (PDF) [file pone.0076835.s002.pdf]

Figure S1

```
L. mono. EGD-e         1 -------MLTHEISENEKPREKLQNYGIEALSSSELVALIIETGTKNESVLTIANRIIMK
L. mono. F2365         1 -------MLIHEISENEKPREKLQNYGIEALSSSELVALIIETGTKNESVLTIANRIIMK
L. mar.                1 -------MLINEVLENEKPREKLQNYGIEALSSSELVALIIETGTKNESVLTIANRIIMK
L. inn.  Clip11262     1 -------MLVNEISENEKPREKLQNYGIEALSTSELVALIIETGTKTESVLTIANRIMMK
L. wel.  SLCC5334      1 -------MLTSEISESEKPREKLQNYGIEALSSSELVALIIETGTKNESVLTIANRIIMK
L. iv.   FSL F6-596    1 -------MLSNEISGNEKPREKLQNYGIEALSTSELVAIIIETGTKSESVLTIANRIIMK
L. seel. SLCC3954      1 -------MLANEISGSEKPREKLQNYGIEALSTSELVAIIIETGTKNESVLTIANRIIMK
L. gr.                 1 -------MQMKEMQIFDKPREKMQTRGQAQLTITELLAILLETGTKEDSVLSLANKIILK
B. smi.  7_3_47FAA     1 --MSKESLRIHNVPREDRPRERLILKGAHSLSNHELIAILLQSGTKEESVLQLANRLLNQ
B. coag. 36D1          1 ----MEKMLIKDFPAEDRPRERLIRTGAESLSNQELLAILLRTGTKEESVLELANRLIRH
B. subt. 168           1 MVIHDLPLKLKDFPMKEKPRERLLKVGAENLANHELLAILLRTGTKHESVLDLSNRLLRS
B. weih. KBAB4         1 ------MNGIRDVVREEQPRERLLLEGAGSLSNRELLAVLLRTGSKEETVLTLSDNILHH
S. aur.  71193         1 -------MKIKEMVTSEMPRERLLSHGAKSLSNTELLAILINTGRKGFSSIDISNELLKS
S. aur.  COL           1 -------MKIKEMVTSEMPRERLLSHGAKSLSNTELLAILINTGRKGFSSIDISNELLKS
E. faec. DO            1 ----MGKRLIKEVPTSSLPRERMEIYGAEALSDQELLAILLRTGQHPYSVMSIAGNLLKT
consensus              1        ......   ..***... .* ..*. .**.*.....*.. .............

L. mono. EGD-e        54 FKNVGEMQYASIEEFQLVNGIGIAKASKIMAAIELGRRI--SIVTEQEEVVVRCPEDAVK
L. mono. F2365        54 FKNVGEMQYASIEEFQLVNGIGIAKASKIMAAIELGRRI--GIVTEQEEVVVRCPEDAVK
L. mar.               54 FKNVAEMQYASIEEFQLVNGIGIAKASKIMAAVELGRRI--SLVTEQEEVVVRCPEDAVK
L. inn.  Clip11262    54 FKHIAEMQYASIEEFQLVNGIGIAKASKIMAAVELGKRI--SLVTEQKEIVIRCPEDAVK
L. wel.  SLCC5334     54 FKHVAEMQYASIEEFQLVNGIGVAKASKIMAAIELGRRI--SIVTNQEEIVIRCPDDAVK
L. iv.   FSL F6-596   54 FKHVAEMQYASLEEFQLINGIGIAKASKIMAAIELGKRI--SLVTEREEIVIRCPGDAAK
L. seel. SLCC3954     54 FKHVAEMQYASLEELQLINGIGLAKASKIMAAVELGKRI--SLVTEREEIVIRCPDDAVK
L. gr.                54 YKSLGDIKDLTIEELRSINGIGLAKATKIMAAIEIGKRICLQSESEKNNIAIRSPQDAVS
B. smi.  7_3_47FAA    59 FEGLRMLKDATLEEITSIKGIGRAKAVQIMAALELGRRI--HSLSLDERYVIRSPEDGAN
B. coag. 36D1         57 FEGLRFLKDASLEEMTSIKGIGTAKAVQILAAIELGRRI--ANLQHDTRYVIRTPQDGAN
B. subt. 168          61 FDGLRLLKEASVEEISSIPGIGMVKAIQILAAVELGSRI--HKLANEEHFVIRSPEDGAN
B. weih. KBAB4        55 FDGLRMLKDATLEEMMSIHGVGIAKASQLMAAFELGRRM--VRLEYQNRYSIRSPEDCAS
S. aur.  71193        54 ASNLNELKKSSINDLIQVKGIGLQKAITLKAAFELGERM--GRRAENNRIKITQPSDVAD
S. aur.  COL          54 ASNLNELKKSSINDLIKVKGIGLQKAITLKAAFELGERM--GRRAENNRIKITQPSDVAD
E. faec. DO           57 FGGLASLRQATLHELEEIRGIGRVKAIEIKALIELGRRI--QTDHERAAPPVRTSYELAQ
consensus             61 .. ..... ......  ..*.*..**....*..*.*.*.    ..... .... .....

L. mono. EGD-e       112 LVMPELAFLFQEHFHCLFLNTKNQVIYRQTIFVGGLNASIVHPREVFRLALRKSAASIMC
L. mono. F2365       112 LVMPELAFLFQEHFHCLFLNTKNQVIYRQTIFVGGLNASIVHPREVFRLALRKSAASIMC
L. mar.              112 LVMPELAFLFQEHFHCLFLNTKNQVIYRQTIFVGGLNASIVHPREVFRLALRKSAASIMC
L. inn.  Clip11262   112 LVMPELAFLFQEHFHCIFLNTKNQVIYRQTIFVGGLNASIVHPREVFRLALRKSSASIMC
L. wel.  SLCC5334    112 LVMPELAFLFQEHFHCIFLNTKNQVIYRQTIFVGGLNASIVHPREVFRLALRKSSAAIMC
L. iv.   FSL F6-596  112 LVMPELAFLFQEHFHCIFLNTKNQVIYRQTIFVGGLNASIVHPREVFRLALRKSAASIMC
L. seel. SLCC3954    112 LVMPELAFLFQEHFHCIFLNTKNQVIYRQTIFVGGLNASIVHPREVFRLALRKSAASLMC
L. gr.               114 IVLPELAFLFQEHFHCVLLNTRNQIVHRQTIFIGSLNASIVHPREVYGLAVRKSAAQIMC
B. smi.  7_3_47FAA   117 YVMQEMRFLTQEHFVCLFLNTKNQVLHKQTIFIGSLNASIVHPREVFKEALRRSAASIIC
B. coag. 36D1        115 YVMNDMRFLSQEHFVCLYLNIKNQVIHRQTIFIGSLNASIVHPREVFKEALRRSAASIIC
B. subt. 168         119 LVMEDMRFLTQEHFVCLYLNTKNQVIHKRTVFIGSLNSSIVHPREVFKEAFKRSAASFIC
B. weih. KBAB4       113 YMMEEMRFLQQEHFVCLYLNTKNQVIHRQTIFIGSLNTSIVHPREVFKEAFRRAAASIIC
S. aur.  71193       112 YMIPTMKDLTQEHFVILLLNSKNVVIKETCVFKGTLNSSIVHPREIFSIAVRENANAIIA
S. aur.  COL         112 YMIPTMKDLTQEHFVILLLNSKNVVIKETCVFKGTLNSSIVHPREIFSIAVRENANAIIA
E. faec. DO          115 QLILEMKDHKQEHLVCIYLDTKNQVILKKTVFIGSLNQSVAHPREIFHYAVRYCAARIVL
consensus            121 ..........***.....*...*... ....*.*.**.*..****....*..........
```

```
L. mono. EGD-e       172 FHNHPSGDP-TPSSEDLLVTKRLAEAGNIVGITLLDHIIIGKNKYISLKEKGYF----
L. mono. F2365       172 FHNHPSGDP-TPSSEDLLVTKRLAEAGNIVGITLLDHIIIGKNKYISLKEKGYF----
L. mar.              172 FHNHPSGDP-APSSEDLLVTKRLAEAGNIVGITLLDHIIIGKNKYISLKEKGYF----
L. inn.  Clip11262   172 FHNHPSGDP-APSSEDLLVTKRLAEAGNIVGITLLDHIIIGKNKYISLKEKGYF----
L. wel.  SLCC5334    172 FHNHPSGDP-TPSSEDLLVTKRLVEAGNIIGITLLDHIIIGKNKYISLKEKGYF----
L. iv.   FSL F6-596  172 FHNHPSGDP-TPSSEDLLVTKRLVEAGSIIGITLLDHIIIGKNKYISLKEKGYF----
L. seel. SLCC3954    172 FHNHPSGDP-SPSSEDLLVTKRLVEAGNIIGITLLDHIIIGKNKYISLKEKGYF----
L. gr.               174 FHNHPSGDP-TPSPEDIQVTKNLAKAGDIIGIPLIDHIIIGKGSFTSLKEQGYF----
B. smi.  7_3_47FAA   177 FHNHPSGDP-TPSREDIEVTKRLAECGKIIGIEVLDHLIIGDQKYISLKEKGYL----
B. coag. 36D1        175 FHNHPSGDP-SPSKEDIEVTKRLAECGKIMGIEILDHLIIGEKKFVSLRQKGYL----
B. subt. 168         179 VHNHPSGDP-TPSREDIEVTRRLFECGNLIGIELLDHLVIGDKKFVSLKEKGYL----
B. weih. KBAB4       173 LHNHPSGDP-APSREDIEVTKRLVECGRIIGIEVLDHIIIGDHKFVSLKEKGHI----
S. aur.  71193       172 VHNHPSGDV-TPSQEDIITTMRLKECGLILGIDLLDHIIIGDNRFTSLVEAGYFDEND
S. aur.  COL         172 VHNHPSGDV-TPSQEDIITTMRLKECGLILGIDLLDHIIIGDNRFTSLVEAGYFDEND
E. faec. DO          175 AHNHPSGNV-IPSQQDMNFTKRIQKCGEMMGITVLDHLIIGRKRYFSLREEGMMEEK-
consensus            181 .******... ** .*...*... ..* ..**...**..**.....**...*..
```

Figure S1: Amino acid based alignment of RadC proteins from various *Firmicutes*.

The alignment was done with MAFFT (Katoh K, Toh H (2008), Brief Bioinform 9: 286-298), shading of conserved amino acid residues was performed with Boxshade. The insertion site of Tn*6188* and other Tn*554*-like transposons is highlighted in red. The consensus is displayed at the bottom of each alignment block, asterisks indicate identical positions, dots indicate similar positions.

Abbreviations and accession numbers: *L. mono.* EGD-e (*L. monocytogenes* EGD-e, NP_465074), *L. mono.* F2365 (*L. monocytogenes* F2365, YP_014167), *L. mar.* (*L. marthii*, WP_008947916), *L. inn.* Clip11262 (*L. innocua* Clip11262, NP_470920), *L. wel.* SLCC5334 (*L. welshimeri* SLCC5334, YP_849759), *L. iv.* FSL F6-596 (*L. ivanovii* FSL F6-596, ZP_07873944), *L. seel.* SLCC3954 (*L. seeligeri* SLCC3954, YP_003464701), *L. gr.* (*L. grayi*, WP_003755617), *B. smi.* 7_3_47FAA (*Bacillus smithii* 7_3_47FAA, ZP_09354272), *B. coag.* 36D1 (*Bacillus coagulans* 36D1, YP_004860597), *B. subt.* 168 (*Bacillus subtilis* 168, NP_390682), *B. weih.* KBAB4 (*Bacillus weihenstephanensis* KBAB4, YP_001647086), *S. aur.* 71193 (*Staphylococcus aureus* 71193, AFH69892), *S. aur.* COL (*Staphylococcus aureus* COL, YP_186546), *E. faec.* DO (*Enterococcus faecium* DO, AFK59806)
