# Supplementary material for: Tn6188 - A Novel Transposon in Listeria monocytogenes Responsible for Tolerance to Benzalkonium Chloride
Source: PLoS One. 2013 Oct 2;8(10):e76835. doi: 10.1371/journal.pone.0076835 (PMC3788773; doi:10.1371/journal.pone.0076835)
Supplement: Figure S2 — Nucleic acid based alignment of radC sequences from various Firmicutes. (PDF) [file pone.0076835.s003.pdf]

Figure S2

```
L. mono. EGD-e      1   ---------------------ATGTTGACACATGAAATATCAGAGAATGAAAAACCACGC
L. mono. F2365      1   ---------------------ATGTTGATACATGAAATATCAGAGAATGAAAAACCACGC
L. mar.  FSL S4-120 1   ---------------------ATGTTAATTAATGAAGTTCTGGAAAATGAAAAACCGCGT
L. inn.  Clip11262  1   ---------------------ATGCTAGTAAATGAAATCTCGGAAAATGAGAAGCCACGA
L. wel.  SLCC5334   1   ---------------------ATGCTTACAAGTGAGATTTCAGAAAGCGAAAAACCACGT
L. iv.   FSL F6-596 1   ---------------------ATGTTATCAAACGAAATTTCAGGAAATGAGAAGCCTCGC
L. seel. SLCC3954   1   ---------------------ATGCTAGCAAACGAAATTTCAGGGAGCGAAAAGCCCCGT
L. gr.   DSM20601   1   ---------------------ATGCAAATGAAAGAAATGCAGATTTTTGATAAACCTCGA
B. smi.  7_3_47FAA  1   ------TTGTCAAAAGAATCGCTTAGGATCCATAACGTTCCACGTGAAGATCGGCCGCGG
B. coag. 36D1       1   ------------TTGGAAAAAATGCTGATCAAAGATTTTCCTGCAGAAGACCGCCCGCGT
B. subt. 168        1   TTGGTCATACACGATCTGCCATTAAAACTCAAAGATTTCCCAATGAAAGAAAAGCCAAGG
B. weih. KBAB4      1   ------------------ATGAACGGTATTCGTGATGTTGTGAGAGAAGAACAGCCACGG
S. aur.  COL        1   ---------------------TTGAAAATTAAAGAAATGGTAACTTCAGAAATGCCAAGA
S. aur.  71193      1   ---------------------TTGAAAATTAAAGAAATGGTAACTTCAGAAATGCCAAGA
E. faec. DO         1   ------------TTGGGAAAACGATTGATCAAAGAAGTACCTACTAGCTCTTTACCAAGG
consensus           1                        ... .... .. .*..*  ....... ......**..*.

L. mono. EGD-e      40  GAAAAACTTCAGAATTATGGTATAGAAGCACTTTCATCTTCGGAACTGGTTGCTTTAATT
L. mono. F2365      40  GAAAAGCTACAGAATTATGGGATAGAAGCACTTTCATCTTCGGAATTGGTTGCTTTAATT
L. mar.  FSL S4-120 40  GAAAAGTTACAGAATTATGGGATAGAGGCTCTTTCTTCTTCGGAGTTAGTTGCTTTAATT
L. inn.  Clip11262  40  GAAAAATTACAAAATTATGGGATAGAGGCGCTTTCGACTTCGGAGTTAGTGGCATTAATT
L. wel.  SLCC5334   40  GAAAAACTTCAAAATTATGGGATAGAGGCTCTTTCGTCATCGGAATTAGTCGCGTTAATA
L. iv.   FSL F6-596 40  GAGAAATTACAAAACTATGGTATAGAGGCACTTTCTACTTCAGAATTAGTCGCAATTATC
L. seel. SLCC3954   40  GAAAAGCTACAAAATTATGGCATTGAAGCACTTTCAACGTCAGAGCTTGTTGCGATAATA
L. gr.   DSM20601   40  GAAAAAATGCAGACAAGAGGGCAAGCGCAACTTACCATAACGGAACTTCTTGCGATATTA
B. smi.  7_3_47FAA  55  GAACGACTGATATTAAAAGGAGCGCACAGCTTGTCCAACCATGAATTGATCGCCATTCTT
B. coag. 36D1       49  GAACGGTTGATCCGCACAGGAGCGGAGAGCCTCTCCAATCAGGAACTGCTTGCCATTTTA
B. subt. 168        61  GAACGGCTCCTGAAAGTCGGAGCCGAGAACTTAGCGAATCATGAACTTTTGGCTATATTA
B. weih. KBAB4      43  GAGCGTTTATTGTTAGAAGGAGCAGGAAGTTTATCGAATCGAGAACTTCTTGCAGTTTTA
S. aur.  COL        40  GAACGTTTGTTAAGCCATGGTGCAAAAAGTCTTTCGAATACAGAATTATTAGCTATATTA
S. aur.  71193      40  GAACGTTTGTTAAGCCATGGTGCAAAAAGTCTTTCGAATACAGAATTATTAGCTATATTA
E. faec. DO         49  GAACGTATGGAAATTTATGGCGCGGAAGCTTTATCGGATCAGGAGCTTTTGGCAATCTTA
consensus           61  **.... *...... ...**.. ...... .*..*.. . ..**..*..* ** .*. *.

L. mono. EGD-e      100 ATTGAAACAGGAACTAAAAATGAGTCAGTTTTGACGATAGCTAATCGAATCATCATGAAG
L. mono. F2365      100 ATTGAAACAGGGACTAAAAATGAGTCAGTTTTGACGATAGCTAATCGAATCATCATGAAA
L. mar.  FSL S4-120 100 ATTGAAACTGGAACGAAAAATGAGTCTGTTTTAACAATAGCAAATCGAATTATTATGAAA
L. inn.  Clip11262  100 ATTGAAACTGGAACGAAAACGGAATCAGTTTTAACAATTGCTAATCGAATTATGATGAAA
L. wel.  SLCC5334   100 ATTGAAACAGGCACAAAAAATGAATCGGTTTTAACAATAGCAAATAGAATTATCATGAAA
L. iv.   FSL F6-596 100 ATTGAAACAGGCACGAAAAGTGAGTCTGTTTTAACTATCGCGAACCGTATTATTATGAAA
L. seel. SLCC3954   100 ATTGAAACAGGAACGAAAAATGAGTCTGTTTTGACAATTGCTAATCGGATTATTATGAAG
L. gr.   DSM20601   100 TTGGAAACGGGAACGAAAGAAGATTCCGTTTTATCATTAGCCAATAAAATCATTCTTAAA
B. smi.  7_3_47FAA  115 CTTCAGTCGGGGACAAAAGAAGAGTCCGTCTTGCAGCTGGCAAATCGGCTATTAAATCAG
B. coag. 36D1       109 CTCCGTACCGGCACGAAAGAAGAATCGGTGCTCGAGCTTGCCAACAGACTGATCCGCCAT
B. subt. 168        121 TTGCGGACAGGGACTAAACACGAATCTGTTTTGGACCTGTCAAACCGGCTGCTGCGCTCA
B. weih. KBAB4      103 CTCAGAACGGGTTCTAAAGAAGAAACGGTGTTAACGTTATCAGATAATATTCTCCATCAT
S. aur.  COL        100 ATTAACACCGGAAGAAAAGGATTCTCGAGCATAGACATTAGTAATGAACTGCTTAAATCT
S. aur.  71193      100 ATTAACACCGGAAGAAAAGGATTCTCGAGTATTGACATTAGTAATGAACTGCTTAAATCT
E. faec. DO         109 CTGCGTACCGGTCAACACCCTTATAGTGTGATGTCGATTGCTGGAAATCTGTTAAAAACG
consensus           121 .*.....*.**.....*... ..........*.....*... .......* .* ......
```

```
L. mono. EGD-e       160 TTCAAAAATGTGGGTGAAATGCAATATGCTTCTATAGAAGAATTTCAATTAGTTAATGGT
L. mono. F2365       160 TTCAAAAACGTGGGCGAAATGCAATATGCTTCTATAGAAGAATTTCAGTTAGTTAATGGT
L. mar.  FSL S4-120  160 TTCAAAAATGTGGCAGAAATGCAGTATGCTTCTATTGAAGAATTTCAATTAGTTAATGGT
L. inn.  Clip11262   160 TTTAAACATATAGCTGAAATGCAGTACGCGTCTATTGAAGAATTTCAATTAGTGAATGGA
L. wel.  SLCC5334    160 TTTAAGCATGTAGCAGAAATGCAATATGCTTCAATCGAGGAATTCCAACTTGTGAATGGA
L. iv.   FSL F6-596  160 TTCAAACATGTAGCTGAAATGCAATATGCTTCTTTGGAAGAGTTTCAATTAATTAATGGC
L. seel. SLCC3954    160 TTTAAACATGTAGCAGAAATGCAATATGCTTCTTTAGAAGAACTTCAATTAATTAACGGA
L. gr.   DSM20601    160 TATAAATCGCTAGGAGATATCAAAGATCTGACTATCGAAGAATTGCGATCTATCAATGGT
B. smi.  7_3_47FAA   175 TTCGAAGGATTACGAATGTTAAAAGATGCTACTTTAGAAGAAATTACCTCTATTAAAGGA
B. coag. 36D1        169 TTTGAAGGCTTGCGGTTTTTAAAAGATGCTTCCCTTGAGGAAATGACGTCCATCAAAGGC
B. subt. 168         181 TTTGACGGGCTGCGCCTGCTCAAGGAAGCATCGGTTGAAGAGCTGTCAAGCATCCCGGGA
B. weih. KBAB4       163 TTTGACGGCTTACGAATGTTAAAGGATGCAACGTTAGAAGAGATGATGAGCATTCATGGT
S. aur.  COL         160 GCTTCGAATCTGAATGAATTGAAAAAATCTTCAATTAACGATTTGATAAAAGTTAAAGGG
S. aur.  71193       160 GCATCGAATCTGAATGAATTGAAAAAATCTTCAATTAACGATTTGATACAAGTTAAAGGG
E. faec. DO          169 TTTGGAGGACTGGCCTCTTTACGACAGGCTACTTTGCATGAGTTAGAAGAGATTCGAGGG
consensus            181 ......... *... ....*.... *.....*..* .*.**..* .... ..*....**.

L. mono. EGD-e       220 ATTGGAATAGCAAAAGCTTCTAAAATCATGGCTGCGATTGAACTAGGAAGGCGTATCAGC
L. mono. F2365       220 ATCGGAATAGCAAAAGCTTCTAAAATCATGGCTGCGATTGAACTTGGAAGACGTATCGGC
L. mar.  FSL S4-120  220 ATCGGAATAGCTAAAGCATCTAAAATTATGGCTGCAGTTGAACTTGGTAGGCGTATAAGT
L. inn.  Clip11262   220 ATTGGAATTGCTAAAGCCTCAAAAATTATGGCAGCAGTCGAACTTGGAAAACGCATCAGT
L. wel.  SLCC5334    220 ATTGGAGTAGCAAAAGCCTCAAAAATAATGGCAGCAATTGAGCTGGGTAGGCGTATTAGT
L. iv.   FSL F6-596  220 ATTGGTATTGCTAAAGCTTCTAAAATAATGGCCGCTATTGAATTAGGAAAGAGAATTAGT
L. seel. SLCC3954    220 ATAGGGCTTGCCAAGGCTTCAAAAATAATGGCAGCAGTAGAATTAGGGAAACGAATTAGC
L. gr.   DSM20601    220 ATTGGACTTGCGAAAGCTACTAAAATCATGGCTGCGATCGAGATTGGGAAACGGATCTGT
B. smi.  7_3_47FAA   235 ATTGGTCGGGCGAAAGCTGTTCAAATCATGGCGGCGCTGGAACTGGGCAGACGTATCCAC
B. coag. 36D1        229 ATCGGCACAGCAAAAGCGGTCCAGATTCTGGCCGCGATTGAACTCGGCAGAAGGATTGCC
B. subt. 168         241 ATCGGTATGGTAAAAGCGATTCAAATACTGGCAGCAGTTGAGCTTGGAAGCCGGATTCAT
B. weih. KBAB4       223 GTTGGGATTGCAAAGGCGTCGCAGCTTATGGCTGCTTTTGAATTAGGTAGAAGAATGGTA
S. aur.  COL         220 ATTGGATTACAAAAAGCAATTACTTTAAAAGCAGCATTTGAGTTAGGGGAAAGGATGGGG
S. aur.  71193       220 ATTGGATTACAAAAAGCAATTACATTAAAAGCGGCATTTGAGTTAGGGGAAAGGATGGGG
E. faec. DO          229 ATAGGACGCGTGAAAGCAATCGAAATCAAAGCCTTGATCGAATTAGGTCGACGAATTCA-
consensus            241 .*.**.......**.** .......* ...** ....*.**..*.**.....*.** ..

L. mono. EGD-e       280 ATCGTAACGGAGCAGGAAGAA------GTGGTTGTTAGGTGTCCCGAAGATGCAGTAAAA
L. mono. F2365       280 ATCGTAACGGAGCAGGAAGAA------GTGGTAGTTAGGTGTCCCGAAGATGCGGTAAAA
L. mar.  FSL S4-120  280 TTAGTAACAGAACAAGAGGAG------GTTGTTGTTAGGTGCCCTGAAGATGCGGTTAAA
L. inn.  Clip11262   280 TTAGTTACGGAACAAAAAGAA------ATTGTCATTAGATGTCCGGAAGATGCCGTGAAA
L. wel.  SLCC5334    280 ATTGTAACTAATCAAGAAGAA------ATTGTTATTAGATGCCCTGATGACGCGGTGAAA
L. iv.   FSL F6-596  280 TTAGTAACGGAGCGTGAAGAA------ATTGTCATTAGATGTCCAGGGGATGCAGCGAAA
L. seel. SLCC3954    280 TTAGTGACAGAGCGCGAAGAA------ATTGTAATAAGATGCCCTGACGATGCAGTAAAA
L. gr.   DSM20601    280 CTGCAAAGTGAATCAGAAAAAAATAATATAGCGATCAGAAGTCCACAAGATGCAGTGTCC
B. smi.  7_3_47FAA   295 AGTCTTTCATTAGACGAACGG------TATGTTATCCGCTCTCCCGAAGACGGTGCAAAT
B. coag. 36D1        289 AATTTGCAGCACGATACACGT------TATGTAATCCGCACGCCGCAAGACGGGGCCAAT
B. subt. 168         301 AAATTAGCCAACGAAGAACAT------TTCGTTATTCGCTCCCCGGAAGACGGCGCTAAT
B. weih. KBAB4       283 CGTTTAGAATATCAAAATAGA------TATAGTATTCGAAGTCCAGAAGATTGTGCGAGT
S. aur.  COL         280 AGAAGAGCTGAAAATAATCGT------ATAAAAATAACGCAACCAAGTGATGTTGCTGAT
S. aur.  71193       280 AGAAGAGCTGAAAATAATCGT------ATAAAAATAACGCAACCAAGTGATGTTGCTGAT
E. faec. DO          288 -----GACGGATCATGAAAGAGCGGCTCCACCGGTTCGTACCAGCTACGAATTGGCCCAG
consensus            301 ....................       ..... .*...... ......**....*.....
```

```
L. mono. EGD-e      334 CTTGTAATGCCAGAGCTTGCTTTTCTGTTTCAAGAACATTTCCATTGCCTATTTTTAAAT
L. mono. F2365      334 CTTGTAATGCCAGAGCTTGCTTTTCTTTTTCAAGAACATTTCCATTGTCTATTTTTAAAT
L. mar.  FSL S4-120 334 TTGGTAATGCCTGAGTTAGCGTTTCTTTTTCAAGAACATTTCCACTGCCTCTTTTTAAAC
L. inn.  Clip11262  334 CTAGTAATGCCTGAGTTAGCTTTCCTTTTTCAAGAACATTTCCACTGTATTTTTTTAAAC
L. wel.  SLCC5334   334 TTAGTAATGCCTGAATTAGCTTTTTGTTTCAAGAACATTTTCACTGTATTTTTCTAAAT
L. iv.   FSL F6-596 334 TTAGTTATGCCGGAATTAGCTTTTCTTTTTCAAGAACATTTTCACTGCATTTTTTTAAAT
L. seel. SLCC3954   334 TTAGTTATGCCAGAATTAGCATTTCTATTTCAAGAACATTTTCACTGCATTTTTCTAAAT
L. gr.   DSM20601   340 ATTGTTTTGCCAGAGCTTGCTTTTCTTTTTCAAGAACATTTTCATTGCGTGCTTTTGAAT
B. smi.  7_3_47FAA  349 TATGTCATGCAAGAAATGCGGTTTTTAACACAAGAACATTTCGTGTGCCTCTTCCTTAAC
B. coag. 36D1       343 TATGTCATGAATGATATGCGCTTTTTATCCCAGGAGCATTTTGTCTGCCTGTATTTAAAT
B. subt. 168        355 CTTGTCATGGAGGATATGCGCTTTTTAACCCAGGAGCATTTTGTCTGTTTATACTTAAAT
B. weih. KBAB4      337 TATATGATGGAAGAGATGCGTTTTTTGCAACAGGAGCATTTTGTTTGTTTATATTTGAAT
S. aur.  COL        334 TATATGATTCCAACAATGAAAGATTTAACACAAGAACATTTTGTCATTTTATTGTTAAAT
S. aur.  71193      334 TATATGATTCCAACAATGAAAGATTTAACACAAGAACATTTTGTCATTTTATTGTTAAAT
E. faec. DO         343 CAATTGATTCTGGAAATGAAAGATCACAAACAAGAACATTTGGTTTGTATTTATTTGGAC
consensus           361 ....*..*....... *... .........**.**.*****....... *.....*..*.

L. mono. EGD-e      394 ACCAAAAATCAAGTAATATATCGGCAAACGATTTTTGTTGGTGGTCTGAATGCTTCCATC
L. mono. F2365      394 ACCAAAAACCAAGTAATATATCGGCAAACGATTTTTGTTGGTGGTCTGAATGCTTCCATC
L. mar.  FSL S4-120 394 ACAAAGAATCAAGTGATTTATCGACAAACAATCTTTGTTGGTGGACTGAATGCGTCAATC
L. inn.  Clip11262  394 ACCAAGAATCAAGTGATTTATAGGCAAACAATCTTTGTCGGTGGTTTGAATGCTTCCATT
L. wel.  SLCC5334   394 ACTAAGAATCAAGTCATTTATAGGCAAACTATTTTTGTTGGAGGATTAAATGCATCGATT
L. iv.   FSL F6-596 394 ACTAAAAATCAAGTCATTTACAGACAAACGATTTTTGTTGGGGGACTAAATGCATCTATT
L. seel. SLCC3954   394 ACAAAAAATCAAGTAATCTATAGACAAACAATTTTCGTTGGTGGATTAAATGCATCCATC
L. gr.   DSM20601   400 ACACGAAATCAGATTGTTCATCGTCAGACTATCTTTATCGGCAGTTTGAATGCTTCTATT
B. smi.  7_3_47FAA  409 ACCAAAAATCAAGTTCTCCACAAACAAACCATTTTTATCGGTAGTTTGAATGCTTCGATT
B. coag. 36D1       403 ATTAAAAACCAGGTGATCCACCGGCAGACGATTTTCATCGGAAGCTTAAATGCCTCGATC
B. subt. 168        415 ACAAAAAATCAAGTCATCCATAAACGCACCGTATTTATCGGAAGCCTGAATTCATCTATT
B. weih. KBAB4      397 ACGAAAAATCAAGTTATACATAGGCAAACTATTTTTATTGGAAGTTTAAATACGTCGATT
S. aur.  COL        394 TCAAAAAATGTAGTGATTAAAGAAACCTGTGTTTTTAAGGTACATTAAATAGTTCGATT
S. aur.  71193      394 TCAAAAAATGTAGTGATTAAAGAAACCTGTGTTTTTAAGGTACATTAAATAGTTCGATT
E. faec. DO         403 ACGAAGAATCAAGTGATCTTAAAAAAAACAGTGTTCATCGGTTCGCTGAATCAGTCAGTC
consensus           421 ......**.....*..* ............*.**... **... .*.***.. **..*.

L. mono. EGD-e      454 GTTCACCCTAGAGAAGTTTTTAGATTGGCGCTCAGAAAATCAGCAGCGTCAATTATGTGC
L. mono. F2365      454 GTTCATCCTAGAGAAGTTTTTAGATTAGCGCTCAGGAAATCAGCAGCATCAATCATGTGC
L. mar.  FSL S4-120 454 GTTCATCCAAGAGAAGTGTTTAGATTAGCGCTAAGAAAATCAGCTGCATCTATTATGTGT
L. inn.  Clip11262  454 GTTCATCCTAGAGAAGTTTTTAGACTGGCATTAAGAAAATCATCAGCTTCTATAATGTGT
L. wel.  SLCC5334   454 GTTCATCCGAGAGAGGTATTTAGATTAGCACTTAGAAAGTCCTCAGCAGCTATTATGTGT
L. iv.   FSL F6-596 454 GTCCATCCTAGAGAAGTTTTTAGGTTAGCATTAAGAAAGTCTGCTGCTTCTATCATGTGC
L. seel. SLCC3954   454 GTTCATCCGAGAGAAGTTTTTAGATTAGCTTTAAGAAAATCAGCTGCGTCTCTTATGTGT
L. gr.   DSM20601   460 GTTCATCCGCGAGAAGTTTATGGATTAGCTGTACGGAAATCTGCCGCCCAAATCATGTGT
B. smi.  7_3_47FAA  469 GTTCATCCTAGGGAAGTGTTTAAAGAAGCGCTTCGCCGCTCCGCTGCCTCCATTATTTGC
B. coag. 36D1       463 GTCCATCCCCGCGAAGTGTTCAAAGAAGCGCTCAGGCGTTCAGCTGCTTCCATTATTTGT
B. subt. 168        475 GTCCACCCGCGAGAGGTGTTTAAAGAAGCGTTTAAACGATCTGCCGCTTCCTTTATCTGT
B. weih. KBAB4      457 GTGCACCCAAGGGAAGTTTTTAAAGAAGCGTTCCGTCGGGCAGCAGCCTCTATTATATGT
S. aur.  COL        454 GTACATCCACGTGAAATTTTTAGTATTGCGGTGAGAGAAAATGCCAATGCAATCATCGCA
S. aur.  71193      454 GTACATCCACGTGAAATTTTTAGTATTGCGGTGAGAGAAAATGCCAATGCAATCATCGCA
E. faec. DO         463 GCTCATCCTAGAGAAATTTTCCATTATGCTGTACGTTATTGTGCAGCAAGGATCGTTTTA
consensus           481 *..**.**..*.**..*.*.........**. *......... .* .. .. .*..*....
```

```
L. mono. EGD-e       514 TTTCATAACCATCCATCTGGCGACCCG-ACTCCTTCTAGTGAAGACTTACTTGTAACAAAA
L. mono. F2365       514 TTTCACAACCATCCATCTGGTGATCCG-ACTCCTTCTAGTGAAGACTTGCTTGTAACAAAA
L. mar.  FSL S4-120  514 TTTCACAACCATCCATCTGGGGACCCA-GCTCCTTCAAGCGAAGACCTACTAGTAACCAAA
L. inn.  Clip11262   514 TTTCATAACCATCCGTCTGGTGATCCC-GCGCCTTCAAGCGAAGATTTACTTGTGACTAAA
L. wel.  SLCC5334    514 TTTCATAATCATCCTTCTGGAGACCCA-ACACCTTCAAGTGAAGATTTACTTGTTACTAAA
L. iv.   FSL F6-596  514 TTTCATAATCATCCTTCTGGGGATCCA-ACTCCATCTAGTGAAGACTTACTTGTGACAAAA
L. seel. SLCC3954    514 TTCCACAACCATCCTTCTGGTGATCCG-TCGCCCTCTAGTGAAGATTTACTTGTAACCAAA
L. gr.   DSM20601    520 TTTCACAATCATCCATCGGGAGATCCA-ACTCCTTCTCCCGAAGACATCCAAGTTACTAAG
B. smi.  7_3_47FAA   529 TTTCACAACCACCCATCCGGTGATCCG-ACGCCTAGCCGAGAAGATATTGAGGTGACCAAA
B. coag. 36D1        523 TTCCATAACCATCCATCCGGAGACCCT-TCTCCTTCCAAAGAAGATATTGAAGTTACCAAA
B. subt. 168         535 GTTCATAATCATCCTTCTGGAGATCCG-ACGCCGAGCAGGGAAGATATTGAAGTGACAAGA
B. weih. KBAB4       517 CTTCATAACCATCCCTCAGGAGATCCG-GCGCCGAGCCGTGAAGATATTGAAGTTACAAAA
S. aur.  COL         514 GTTCATAATCATCCATCCGGTGATGTA-ACGCCCTCACAAGAAGATATCATAACAACAATG
S. aur.  71193       514 GTTCATAATCATCCATCCGGTGATGTA-ACGCCCTCACAAGAAGATATCATAACAACAATG
E. faec. DO          523 GCGCATAATCATCCTTCTGGTAATGTT-ATTCCATCGCAGCAGGATATGAATTTTACCAAA
consensus            541 ...**.**.**.**.**.** .*.... ...**... .. .*.**..*.......** *..

L. mono. EGD-e       574 AGATTAGCTGAAGCTGGCAATATTGTCGGAATTACTTTACTAGATCATATTATCATTGGC
L. mono. F2365       574 AGGTTAGCTGAAGCTGGTAATATTGTCGGAATTACCTTACTAGATCATATTATCATTGGC
L. mar.  FSL S4-120  574 AGATTGGCTGAAGCAGGAAATATTGTTGGAATCACTTTGCTAGATCACATTATCATCGGT
L. inn.  Clip11262   574 CGTTTAGCGGAGGCTGGAAATATTGTTGGAATAACGCTTTTAGACCACATTATTATTGGG
L. wel.  SLCC5334    574 AGATTAGTAGAAGCTGGTAACATAATTGGTATTACATTGTTGGATCACATTATTATTGGT
L. iv.   FSL F6-596  574 CGATTAGTGGAAGCGGGTAGTATTATTGGGATTACACTACTAGACCATATTATTATTGGA
L. seel. SLCC3954    574 CGATTAGTTGAAGCTGGTAATATTATTGGGATTACGTTACTAGACCATATTATTATAGGG
L. gr.   DSM20601    580 AATTTGGCAAAAGCCGGGGACATCATCGGCATTCCCTTGATTGATCATATCATCATTGGC
B. smi.  7_3_47FAA   589 CGTTTAGCTGAGTGTGGAAAAATCATCGGAATTGAAGTTCTCGATCATCTCATTATCGGC
B. coag. 36D1        583 CGGCTTGCCGAATGTGGTAAAATAATGGGGATTGAGATTCTTGACCATTTGATCATCGGT
B. subt. 168         595 CGGCTGTTTGAATGCGGAAACCTGATTGGCATCGAGCTGCTTGACCATTTGGTGATCGGG
B. weih. KBAB4       577 CGATTAGTAGAATGTGGTCGAATTATCGGAATTGAAGTACTTGATCATATCATAATAGGT
S. aur.  COL         574 AGGTTGAAGGAGTGTGGTTTGATTTTAGGGATAGATTTATTGGATCATATTATAATCGGT
S. aur.  71193       574 AGGTTGAAGGAGTGTGGTTTGATTTTAGGGATAGATTTATTGGATCATATTATAATCGGT
E. faec. DO          583 AGGATACAAAAGTGTGGAGAAATGATGGGGATCACTGTACTGGACCACTTGATCATCGGA
consensus            601 ....*.. ..*....**... .*..* **.**.....*..*.**.**..*..* ** **

L. mono. EGD-e       634 AAGAATAAGTATATTAGTTTGAAAGAAAAAGGTTACTTT-------------TAA
L. mono. F2365       634 AAGAATAAGTATATTAGTTTGAAAGAAAAAGGTTACTTT-------------TAA
L. mar.  FSL S4-120  634 AAGAATAAGTACATTAGTCTAAAAGAAAAAGGTTATTTT-------------TAA
L. inn.  Clip11262   634 AAAAACAAATACATTAGTTTAAAAGAAAAAGGCTACTTT-------------TAA
L. wel.  SLCC5334    634 AAAAATAAATATATTAGTCTGAAAGAAAAAGGCTATTTT-------------TAA
L. iv.   FSL F6-596  634 AAAAATAAGTACATTAGTCTAAAAGAAAAAGGTTATTTC-------------TGA
L. seel. SLCC3954    634 AAAAATAAATATATCAGTTTAAAAGAAAAAGGCTATTTT-------------TAA
L. gr.   DSM20601    640 AAGGGTTCATTTACCAGTTTAAAAGAACAGGGATATTTT-------------TAA
B. smi.  7_3_47FAA   649 GACCAAAAATACATTAGTTTAAAAGAAAAAGGCTACTTA-------------TAA
B. coag. 36D1        643 GAGAAAAAATTTGTAAGTCTGAGACAAAAAGGGTATTTG-------------TAA
B. subt. 168         655 GATAAAAAATTTGTGAGTTTAAAGGAAAAAGGATATTTG-------------TAA
B. weih. KBAB4       637 GACCATAAATTCGTGAGTTTAAAGGAAAAAGGTCATATT-------------TAA
S. aur.  COL         634 GATAATAGATTTACCAGTCTTGTAGAAGCGGGTTACTTTGATGAAAATGATTGA
S. aur.  71193       634 GATAATAGATTTACGAGTCTTGTAGAAGCGGGTTACTTTGATGAAAATGATTGA
E. faec. DO          643 CGCAAACGATATTTTAGTTTGAGAGAAGAAGGAATGATGGAA---GAGAAGTAA
consensus            661 .........*.....***.*.....**...** ....*.             *.*
```

Figure S2: Nucleic acid based alignment of *radC* sequences from various *Firmicutes*.
The alignment was done with MAFFT (Katoh K, Toh H (2008), Brief Bioinform 9: 286-298), shading of conserved residues was performed with Boxshade. The insertion site of Tn*6188* and other Tn*554*-like transposons is highlighted in red. The consensus is displayed at the bottom of each alignment block, asterisks indicate identical positions, dots indicate similar positions.
Abbreviations and accession numbers: *L. mono.* EGD-e (*L. monocytogenes* EGD-e, NC_003210), *L. mono.* F2365 (*L. monocytogenes* F2365, NC_002973), *L. mar.* FSL S4-120 (*L. marthii* FSL S4-120, ADXF01000663), *L. inn.* Clip11262 (*L. innocua* Clip11262, NC_003212), *L. wel.* SLCC5334 (*L. welshimeri* SLCC5334, NC_00855), *L. iv.* FSL F6-596 (*L. ivanovii* FSL F6-596, ADXI01000816), *L. seel.* SLCC3954 (*L. seeligeri* SLCC3954, NC_013891), *L. gr.* DSM20601 (*L. grayi* DSM 20601, ACCR02000005), *B. smi.* 7_3_47FAA (*Bacillus smithii* 7_3_47FAA, EHL73379), *B. coag.* 36D1 (*Bacillus coagulans* 36D1, NC_016023), *B. subt.* 168 (*Bacillus subtilis* 168, NC_000964), *B. weih.* KBAB4 (*Bacillus weihenstephanensis* KBAB4, NC_010184), *S. aur.* COL_S. (*Staphylococcus aureus* COL, NC_002951), *S. aur.* 71193 (*Staphylococcus aureus* 71193, AFH69892), *E. faec.* DO (*Enterococcus faecium* DO, AFK59806).
