# Supplementary material for: Tn6188 - A Novel Transposon in Listeria monocytogenes Responsible for Tolerance to Benzalkonium Chloride
Source: PLoS One. 2013 Oct 2;8(10):e76835. doi: 10.1371/journal.pone.0076835 (PMC3788773; doi:10.1371/journal.pone.0076835)
Supplement: Figure S3 — Growth of two L. monocytogenes strains with and without Tn6188 in the presence of different BC concentrations (0-5 mg/l) at 37°C. (PDF) [file pone.0076835.s004.pdf]

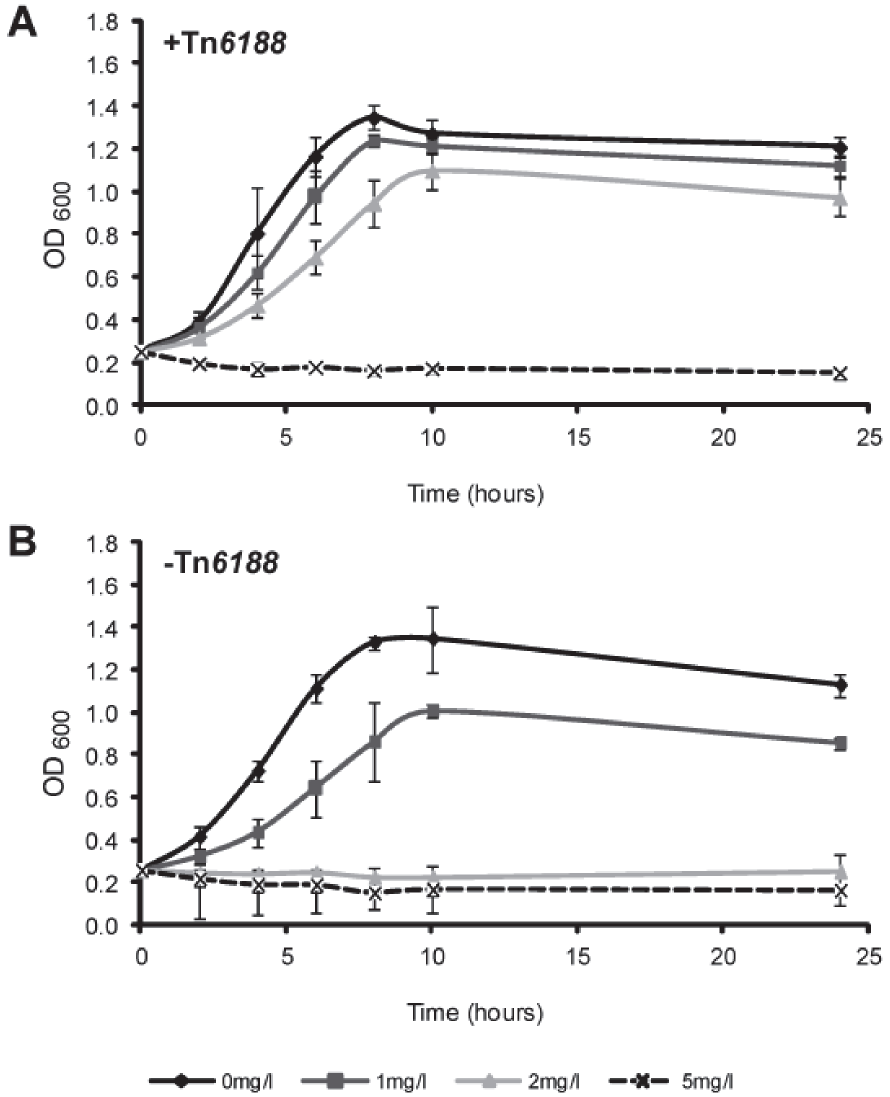

Figure S3: Growth of two *L. monocytogenes* strains with (+Tn*6188*, **A**) and without Tn*6188* (-Tn*6188*, **B**) in the presence of different BC concentrations (0-5 mg/l) at 37°C. Values represent mean values ± SD. More details on the used strains can be found in Table S1. All experiments were performed in two biological independent replicates. Optical density was measured at 600 nm (OD_{600}).
