# Supplementary material for: Tn6188 - A Novel Transposon in Listeria monocytogenes Responsible for Tolerance to Benzalkonium Chloride
Source: PLoS One. 2013 Oct 2;8(10):e76835. doi: 10.1371/journal.pone.0076835 (PMC3788773; doi:10.1371/journal.pone.0076835)
Supplement: Figure S4 — PCR analysis of L. monocytogenes qacH deletion mutants. (PDF) [file pone.0076835.s005.pdf]

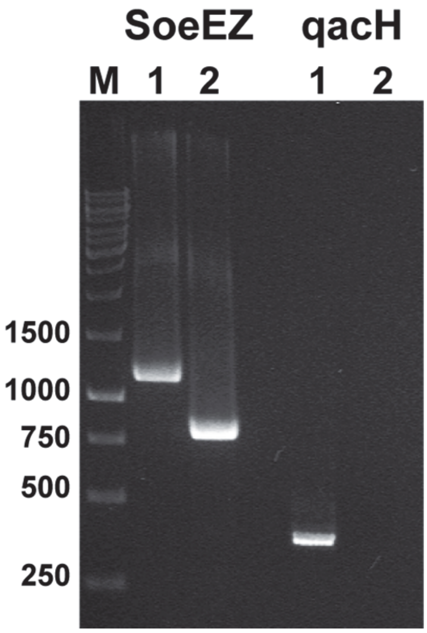

Figure S4: PCR analysis of *L. monocytogenes qacH* deletion mutants. PCR analysis using SoeE and SoeZ primers (SoeEZ) resulted in a 1170 bp (wildtype strain 4423, **1**) or 799 bp fragment (Δ*qacH* strain 4423, **2**). PCR targeting the *qacH* gene resulted in a 366 bp amplicon only in the wildtype strain 4423 (**1**). PCR reactions for *L. monocytogenes* strain 6179 WT and Δ*qacH* showed similar results – data are not shown. M: marker.
