# Supplementary material for: Tn6188 - A Novel Transposon in Listeria monocytogenes Responsible for Tolerance to Benzalkonium Chloride
Source: PLoS One. 2013 Oct 2;8(10):e76835. doi: 10.1371/journal.pone.0076835 (PMC3788773; doi:10.1371/journal.pone.0076835)
Supplement: Figure S5 — Growth of L. monocytogenes 4423 wildtype strain and L. monocytogenes 4423 ΔqacH strain in the presence of different BC concentrations (0-5 mg/l) at 37°C. (PDF) [file pone.0076835.s006.pdf]

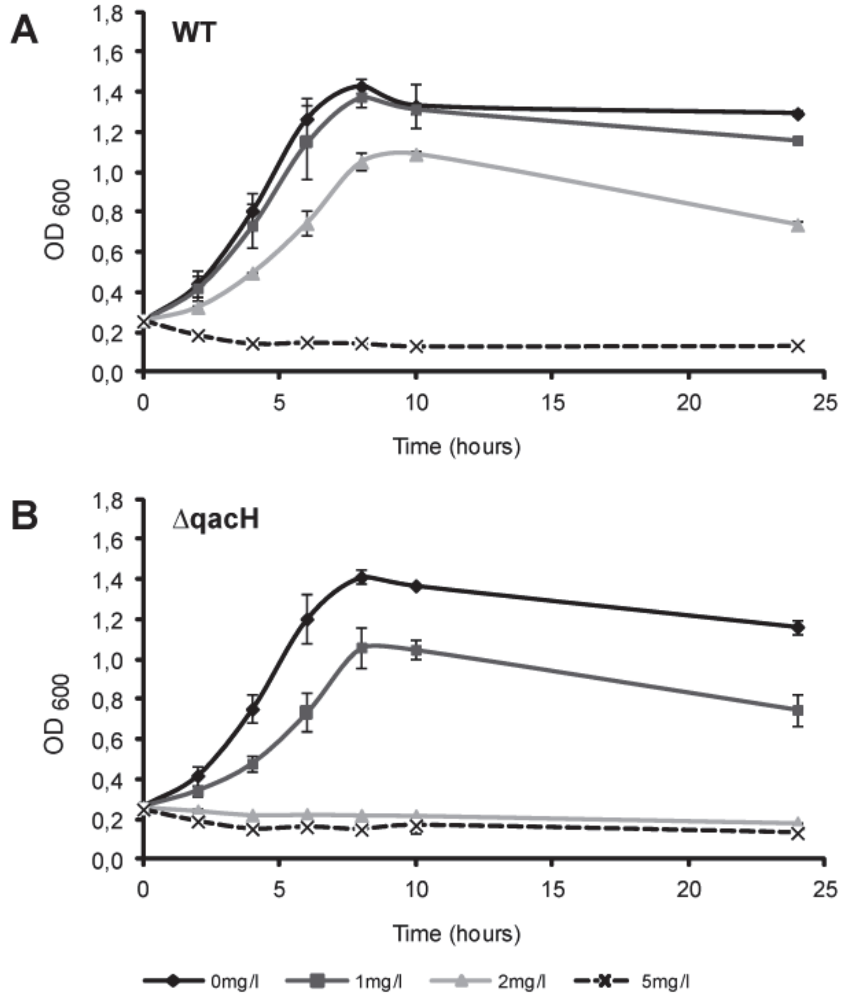

Figure S5: Growth of *L. monocytogenes* 4423 wildtype strain (**A**) and *L. monocytogenes* 4423 Δ*qacH* strain (**B**) in the presence of different BC concentrations (0-5 mg/l) at 37°C. Values represent mean values ± SD. More details on the used strain can be found in Table S1. All experiments were performed in two biological independent replicates. Optical density was measured at 600 nm (OD_{600}). *L. monocytogenes* 6179 Δ*qacH* showed similar growth pattern – data are not shown.
